# As(III)-oxidizing and plant growth-promoting bacteria increase the starch biosynthesis-related enzyme activity, 2-AP levels, and grain quality of arsenic-stressed rice plants

**DOI:** 10.1186/s12870-024-05352-6

**Published:** 2024-07-15

**Authors:** Sarun Thongnok, Wilailak Siripornadulsil, Lalita Thanwisai, Surasak Siripornadulsil

**Affiliations:** 1 Department of Microbiology, Faculty of Science, Khon Kaen University, 123 Mittraphap Road, Nai-Muang, Muang District, Khon Kaen, 40002 Thailand; 2 Research Center for Environmental and Hazardous Substance Management, Khon Kaen University, Khon Kaen, 40002 Thailand; 3 Salt-tolerant Rice Research Group, Khon Kaen University, Khon Kaen, 40002 Thailand

**Keywords:** Arsenic, Starch biosynthesis-related enzyme, As(III)-oxidizing bacteria, Plant growth-promoting bacteria, 2-AP aromatic compound

## Abstract

**Background:**

Grain quality is an important index of rice production, particularly when plants are grown under stress. Arsenic (As) contamination in paddy fields severely affects rice grain yield and quality. Here, the effects of As and combinations of As(III)-oxidizing bacteria (*Pseudomonas stutzeri* 4.25, 4.27, and 4.44) and plant growth-promoting bacteria (*Delftia acidovorans* KKU2500-12 and *Cupriavidus taiwanensis* KKU2500-3) on enzymes related to starch accumulation in grains and the grain quality of Khao Dawk Mali 105 rice cultivated in As-contaminated soil under greenhouse conditions were investigated.

**Results:**

Arsenic affected the activities of starch biosynthesis-related enzymes, and decreases of up to 76.27%, 71.53%, 49.74%, 73.39%, and 47.46% in AGPase, SSS, GBSS, SBE, and SDBE activities, respectively, and 9.42–61.07% in starch accumulation in grains were detected after growth in As-contaminated soil. However, the KKU2500-3/4.25 and KKU2500-3/4.44 combinations yielded the greatest enzyme activities in grains, and compared with the results observed in uninoculated seedlings, increases in starch accumulation of up to 51.16% and 23.81% were found in the inoculated seedlings after growth in medium- and high-As-contaminated soils, at 10–17 and 10–24 days after anthesis, respectively. The bacteria increased the 2-AP content in rice under As stress, possibly via the induction of proline, a 2-AP substrate. Bacterium-inoculated rice had significantly greater 2-AP levels than uninoculated rice, and 2.16–9.93% and 26.57–42.04% increases were detected in rice plants grown in medium- and high-As-contaminated soils, respectively.

**Conclusions:**

Arsenic toxicity can be mitigated in rice growing under greenhouse conditions by maintaining starch biosynthesis, accumulating amylose, and increasing 2-AP content. The effectiveness of these bacteria should be validated in paddy fields; hence, safe rice grains with a good starch content and aroma could be produced.

**Supplementary Information:**

The online version contains supplementary material available at 10.1186/s12870-024-05352-6.

## Highlights

As(III) oxidation and PGPB improved the quality of the KDML 105 rice grains.

Bacteria maintained starch biosynthesis-related enzyme activity in As-contaminated soils.

Bacteria increased starch accumulation and the 2-AP aromatic content in rice grains.

Bacteria improved the crop yield and grain quality of As-stressed rice plants.

**Supplementary Information:**

The online version contains supplementary material available at 10.1186/s12870-024-05352-6.

## Introduction

The problem of arsenic (As) contamination in paddy fields and groundwater is of great concern worldwide because As can adversely affect rice yield and grain quality. As a naturally occurring pollutant, As originates from both natural and anthropogenic activities [1]. Inorganic As, namely, arsenite (the reduced form of As, As(III)) and arsenate (the oxidized form of As, As(V)), are found mainly in nature and are interconvertible depending on the redox conditions in the soil [2]. Rice is a major staple food crop of the global population and is generally grown in flooded soils where high As availability results in rice accumulating As to levels greater than those found in other cereal crop species [3]. Several factors, including the As concentration, As form, rhizosphere properties, irrigation methods, environmental factors, and rice cultivar, affect the amount of As that rice absorbs from the soil [4, 5]. In terms of the amount of arsenic bioaccumulation, the following order was observed: root > basal stem > median stem > apical stem > leaves > grains. Inorganic As, mostly As(III), is the major species of As found in grains [2]. The toxicity of As to plants involves reducing the growth and pigment contents of plants, which leads to decreased productivity. The toxic effects are dependent on the form of As: As(III) is more toxic than As(V) [2]. As(III) reacts with the sulfhydryl (-SH) groups of enzymes, resulting in the inhibition of enzymes and cellular function [6], whereas As(V) inhibits the uptake of phosphate (an analog of As(V)) in plants [7].

The activity of As-resistant bacteria is used to resolve As toxicity and accumulation in rice by changing the form of As in the soil, which affects the availability and toxicity of As [8]. As(III) oxidation is a major process by which As(III)-oxidizing bacteria reduce the toxicity of As; As(III) oxidation involves the oxidation of highly toxic As(III) to the less toxic form As(V), which is taken up by rice in lower amounts. In addition, some microbes can enhance plant growth by both direct and indirect mechanisms [9], including nitrogen (N) fixation, phosphorus (P) solubilization, indoleacetic acid (IAA) and 1-aminocyclopropane-1-carboxylic acid (ACC) deaminase production, plant stress control, antifungal activity, and the induction of systemic resistance [10]. Previous studies have shown that As toxicity reduces growth parameters, pigment contents, and grain yields. The toxic effect on yield occurs via a reduction in the filled-grain percentage, which leads to a decrease in grain weight. In contrast, inoculation of seedlings with the As(III)-oxidizing bacterium *Pseudomonas stutzeri* and the heavy metal-tolerant plant growth-promoting bacteria *Cupriavidus taiwanensis* KKU2500-3 and *Delftia acidovorans* KKU2500-12 reduced As toxicity to growth and productivity, resulting in enhanced rice growth and increased rice yield. The coinoculated bacteria effectively reduced As accumulation in rice grains. Furthermore, they increase grain weight by reducing unfilled grains [11].

Grain yield and quality are important in cereal crop production and are threatened under stressful conditions. Starch is the major component of rice grains, and it generally consists of amylose and amylopectin, which play important roles in grain quality, yield, and palatability [12]. Starch biosynthesis is coordinated by the activities of several enzymes that occur during the ripening period, including those in the milk, dough, and mature stages. The key enzymes involved in starch biosynthesis are ADP-glucose pyrophosphorylase (AGPase), soluble starch synthase (SSS), granule-bound starch synthase (GBSS), starch-branching enzyme (SBE), and starch-debranching enzyme (SDBE) [13]. In rice grains, starch biosynthesis is affected by abiotic stresses such as drought, salinity, and temperature [12, 14, 15]. However, only a few reports have evaluated the activity of these enzymes in developing rice grains under As stress and their ability to mitigate microbial activity. Moreover, the abiotic and biotic stresses that induce the production of aromatic compounds have been well documented [16]. The Khao Dawk Mali (KDML105) cultivar, or “Thai Jasmine rice”, is known as aromatic rice. Aromatic compounds such as 2-acetyl-1-pyrroline (2-AP) contribute significantly to the fragrance of rice [17, 18]. Furthermore, 2-AP is commonly found in the aboveground portions of rice, such as the grain, stem sheath, and leaves. 2-acetyl-1-pyrroline biosynthesis in fragrant rice is influenced by a variety of factors, including genetics, environmental conditions, and crop management practices [19]. There are two pathways for the biosynthesis of 2-AP: nonenzymatic pathways, in which proline, ornithine and glutamate are catalyzed and react with methylglyoxal to form 2-AP, and enzymatic pathways [20]. Luo et al. (2020) [21] reported that the application of exogenous proline induces the regulation of 2-AP biosynthesis in fragrant rice. The biosynthesis of 2-AP has been found in a wide range of plants and rhizobacteria and has been reported to increase the 2-AP level in aromatic rice grains [22, 23]. In our previous study, coinoculated bacteria, namely, *P*. *stutzeri*, *D*. *acidovorans* KKU2500-12, and *C*. *taiwanensis* KKU2500-3, maintained the productivity of KDML105 rice under As toxicity, but the reasons for their effects on rice productivity are unclear [11]. We hypothesized that these bacteria might mitigate As toxicity, resulting in progressive starch biosynthesis-related enzyme activity and enhancing rice grain quality. Thus, in the present study, the following issues were addressed: (i) the activities of starch biosynthesis-related enzymes in uninoculated and bacterium-inoculated rice plants cultivated under non-As- and As-stress conditions and (ii) the 2-AP content and starch accumulation in rice grains under As stress. Taken together, these data will help determine how As affects grain production and quality and how bacteria can mitigate these effects.

## Materials and methods

### Bacterial conditions and preparation

The plant growth-promoting bacteria *D*. *acidovorans* KKU2500-12 and *C*. *Taiwanensis* KKU2500-3 and the As(III)-oxidizing bacterium *P*. *stutzeri* 4.25, 4.27, and 4.44 were isolated from heavy metal-contaminated areas in Tak and Loie Provinces, Thailand, respectively [24, 25]. These strains were subsequently grown in nutrient broth media at 30 °C for 18 h (corresponding to the log phase). The cell pellets were harvested via centrifugation at 12,298 × g for 15 min. Afterward, the cells were resuspended and prepared in normal saline (0.85% w/v NaCl) at 2.5 × 10^6^ colony forming units (CFU)/mL for future colonization of rice seedlings.

### Greenhouse experiments

Khao Dawk Mali 105 rice (*Oryza sativa* L. var. KDML 105) was obtained from the Khon Kaen Rice Research Center, Thailand. The seeds were surface sterilized using 90% ethanol (EtOH) for 3 min, followed by 0.2% (w/v) mercuric chloride (HgCl_2_) for 30 min, after which they were rinsed twice with sterile distilled water. The seeds were germinated for 3–5 days in a dark room. Once germinated, the seeds were colonized with *P. stutzeri* 4.25, 4.27, or 4.44, *D. acidovorans* KKU2500-12 or *C. taiwanensis* KKU2500-3 at 2.5 × 10^6^ CFU/mL (RB, which served as the control) or remained uncolonized (R). The combined treatments included *D*. *acidovorans* KKU2500-12 and *P*. *stutzeri* 4.25 (RB-12/25), *C*. *taiwanensis* KKU2500-3 and *P*. *stutzeri* 4.25 (RB-3/25), *C*. *taiwanensis* KKU2500-3 and *P*. *stutzeri* 4.27 (RB-3/27), and *C*. *taiwanensis* KKU2500-3 and *P*. *stutzeri* 4.44 (RB-3/44). The bacterial cell suspensions were subsequently added to the germinated seeds. The uninoculated and inoculated rice plants were subsequently transplanted to plug trays containing sterile mixed soil (mixtures of soil, burnt rice husks and manure at 7:2:1 w/w). After being transplanted, the plants were maintained for 30 days.

The pot experiments were conducted in accordance with a completely randomized design (CRD) with three replications in a greenhouse. The soil samples used for the pot experiments were collected from different paddy fields in the Wang Saphung district, Loei Province, Thailand; the samples were collected from non, medium (29.91 mg/kg)-, and high (45.11 mg/kg)-As-contaminated soils (Table S1). The soil samples were sterilized by autoclaving (121 °C for 15 min), and 10 kg of soil was added to each pot. To maintain field capacity, the pots were filled with water a day prior to transplantation, and one seedling was planted in each pot. Over the course of 10 days, sufficient water was provided to the pots to meet the field capacity requirements, thus allowing the rice to grow. Until the grain reached the ripening stage, the water level was maintained at 10 cm above the soil surface. The chemical fertilizers N-P_2_O_5_-K_2_O (16-16-8) and urea (46-0-0) were applied twice at a rate of 0.22 g/pot to the soil at 15 days after transplantation (DAT) (tiller stage) and at 45 DAT (heading stage). The rice plants were grown until they were harvested. The grain panicles were collected at 1, 3, 10, 17, and 24 days after anthesis (DAA) to determine the starch biosynthesis enzyme activity, starch content, and amylose content. At harvest, the soluble protein and aromatic compound (2-AP) contents were determined.

### Starch biosynthesis-related enzyme content

Panicles at 1, 3, 10, 17, and 24 DAA were immediately collected and ground into a fine powder in liquid nitrogen for enzyme assays. Crude enzyme extracts were prepared according to the methods of Jeng et al. (2003) [13]. Dehulled grain powder was homogenized at 4 °C with 4.5 mL of extraction buffer consisting of 100 mM HEPES-KOH buffer (pH 7.2), 10 mM magnesium chloride (MgCl_2_), 5 mM DL-dithiothreitol (DTT), and 1 mM (ethylenedinitrilo)tetraacetic acid disodium salt (Na-EDTA). After centrifugation at 12,298 × g for 20 min, the supernatant and precipitate were resuspended in extraction buffer, which constituted a crude enzyme extract, and subsequently used for protein content determination and enzyme assays. All the steps were conducted at 4 °C.

### AGPase activity

AGPase activity was determined according to the methods of Rocher et al. (1989) [26]. Fifty microliters of the supernatant was mixed in 373 µL of reaction mixture consisting of 50 mM HEPES-NaOH, 5 mM Ppi, 6 mM MgCl_2_ and 3 mM DTT and then incubated at 30 °C for 2 min, followed by the addition of 27 µL of 20 mM ADP-glucose. After incubation at 30 °C for 30 min, the mixtures were boiled for 1 min and then centrifuged at 12,298 × g for 10 min. The supernatants were subsequently reacted in 400 µL of 6 mM NADP, 50 mM HEPES-NaOH and 1 µL of 0.08 U PGM plus 0.07 U G6PDH and incubated at 30 °C for 30 min. The reactions were evaluated at 340 nm and expressed as units of enzyme activity per minute per milligram of protein (unit/min/mg protein).

### SSS activity

SSS activity was determined according to the methods of Nishi et al. (2001) [27]; 200 µL of supernatant was mixed with 223 µL of reaction buffer I, which consisted of GAPDH 50 mM HEPES-NaOH, 1.4 mg of amylopectin and 15 mM DTT, and then incubated at 30 °C for 2 min. Then, 27 µL of 20 mM ADP-glucose was added to the reaction mixture. After incubation at 30 °C for 30 min, the mixtures were boiled for 1 min and then centrifuged at 12,298 × g for 10 min. The supernatants were reacted in 400 µL of reaction buffer II, which consisted of 50 mM HEPES-NaOH, 10 mM glucose, 20 mM MgCl_2_, 2 mM NADP, and 1 µL of 1.4 U of hexokinase plus 0.35 U of G6PDH, and then incubated at 30 °C for 10 min. The reactions were evaluated at 340 nm and expressed as units of enzyme activity per minute per milligram of protein (unit/min/mg protein).

### GBSS activity

GBSS activity was determined according to the methods of Fujita et al. (2001) [28]. First, 100 µL of precipitate resuspended in extraction buffer was mixed with 223 µL of reaction buffer I, which consisted of 50 mM HEPES-NaOH and 15 mM DTT, and then incubated at 30 °C for 2 min. Then, 27 µL of 20 mM ADP-glucose was added to the reaction mixture. After incubation at 30 °C for 30 min, the mixtures were boiled for 1 min and then centrifuged at 12,298 × g for 10 min. The supernatants were reacted in 400 µL of reaction buffer II, which consisted of 50 mM HEPES-NaOH, 200 mM potassium chloride (KCl), 10 mM MgCl_2_, 4 mM phosphoenolpyruvate and 1 µL of 1.2 U of pyruvate kinase. After incubation at 30 °C for 30 min, the mixtures were boiled for 1 min and then centrifuged at 12,298 × g for 10 min. The supernatants were reacted in 400 µL of reaction buffer III, which consisted of 50 mM HEPES-NaOH, 10 mM glucose, 20 mM MgCl_2_, 2 mM NADP, and 1 µL of 1.4 U of hexokinase plus 0.35 U of G6PDH, and then incubated at 30 °C for 10 min. The reactions were measured at 340 nm and expressed as units of enzyme activity per minute per milligramof protein (unit/min/mg protein).

### SBE activity

SBE activity was determined according to the methods of Guan and Preiss (1993) [29]; 100 µL of supernatant was mixed in 100 µL of reaction buffer consisting of 50 mM HEPES-NaOH, 5 mM glucose-1-phosphate, 1.25 mM adenosine-5-monophosphate monohydrate, and phosphorylase. After incubation at 30 °C for 30 min, 50 µL of 1 M hydrochloric acid (HCl) was added, followed by the addition of 500 µL of dimethyl sulfoxide (DMSO) and 700 µL of fresh iodine reagent [8 mM iodine (I_2_)/120 mM potassium iodide (KI)]. After incubation at 30 °C for 30 min in the dark, the reactions were measured at 540 nm, and the results are expressed as units of enzyme activity per minute per milligram of protein (unit/min/mg protein).

### SDBE activity

SDBE activity was determined according to the methods of Pan and Nelson (1984) [30], and 10 g of dehulled grain powder was extracted in 10 mL of 0.1 M citrate buffer containing 0.1 mM DTT, followed by centrifugation at 12,298 × g for 10 min. The supernatants were precipitated in 10–60% ammonium sulfate [(NH_4_)_2_SO_4_] and resuspended in 150 µL of 0.1 M citrate buffer. The reactions were started after the addition of 150 µL of 0.1 M citrate buffer containing 0.1 mM DTT and 1 mg of pullulan. After incubation at 30 °C for 30 min, the amount of reducing sugars was measured using the dinitrosalicylic acid (DNS) method [31]. Then, 500 µL of starch slurry was reacted with 500 µL of DNS solution, and the reaction mixture was boiled at 90 °C for 10 min followed by rapid placement in cool water. Afterward, 5 mL of distilled water was added. The reactions were measured at 540 nm. The reducing sugar contents were calibrated via a standard calibration curve of reducing sugars (0.5–2.0 g/L).

### Quality of rice grains

#### Starch content

The starch content in the grains was determined according to the methods of Timabud et al. (2016) [32]. One hundred milligrams of dehulled grain powder was homogenized in 2 mL of distilled water, followed by homogenization in 2 mL of 2 M NaOH. After incubation at room temperature for 30 min, the homogenates were neutrally prepared with 4 mL of 1 N HCl and then diluted with 1:30 distilled water (starch slurry). Forty microliters of the starch slurry was homogenized in 40 µL of 0.1 M phosphate buffer (pH 7.0) and 20 µL of HCl. The solutions were reacted with 100 µL of fresh iodine reagent (5 mM I_2_ and 5 mM KI). Then, the absorbance was measured at 580 nm and calibrated via a standard calibration curve of soluble starch (0.25–1.50 µg/µL).

#### Amylose content

The amylose content was determined according to the methods of Timabud et al. (2016) [32], and 250 µL of starch slurry was reacted in 1 mL of fresh iodine reagent (0.3% I_2_ and 1.5% KI). After incubation at room temperature for 5 min, the reactions were measured at 620 nm and 535 nm. The amylose content was calculated via the following formula:

amylose content = 1.4935 × exp^(2.7029 × A ratio 620/535)^.

#### Aromatic compound content in the grains

The 2-AP content in the rice grains at harvest was measured according to the methods of Timabud et al. (2016) [32] with some modifications. Rice grains were ground into a fine powder in liquid nitrogen in a mortar. Fifty milligrams of powder was immediately transferred to a 10 mL crimp-neck vial that was subsequently sealed and capped with a polytetrafluoroethylene (PTFE)/silicone septum and bimetal crimp cap, respectively. Ten milliliters of the internal standard of 2 ng of 2,4-dimethylpyridine (DMP) in filtered absolute ethanol was added to the sample. The 2-AP content was measured using headspace gas chromatography‒mass spectrometry (GC‒MS). The vials were transferred to a tray of an autosampler and heated in an oven at 120 °C for 15 min. After heating in the oven block, 2 mL of headspace gas was absorbed and injected into the GC‒MS instrument (GCMS-QP2010, Shimadzu, Japan), whose injection temperature was 250 °C. The process was conducted at 50 °C and increased to 120 °C for 14 min. Helium gas was used for the mobile phase, and the flow was maintained at 0.6 mL/min; the split ratio was maintained at 2.0. The ionized sample was detected and identified via MS in selected ion monitoring (SIM) mode. The 2-AP content in the rice samples was calculated according to the relative peak areas of 2-AP and DMP.

#### Soluble protein content in the rice grains

At harvest, protein was extracted according to the methods described by Ferreira et al. (2002) [33], and 0.5% dehulled grain powder was extracted in 2 mL of 50 mM sodium phosphate buffer (pH 7.0) consisting of 10% polyvinylpyrrolidone (PVP). After overnight incubation at 40 °C, the samples were centrifuged at 12,298 × g for 20 min. The protein concentration in the supernatant was measured using Bradford’s reagent. The protein content was calibrated via a standard calibration curve of bovine serum albumin (BSA) (0.05-10.0 mg/L).

### Proline content in the leaves

At harvest, the proline in the leaves was extracted according to the methods described by Abraham et al. (2010) [34], and 0.5 g of fresh leaves was homogenized with 3% (w/v) sulfosalicylic acid and centrifuged at 12,298 × g for 10 min. Two hundred microliters of supernatant was reacted in 400 µL of the ninhydrin reagent mixture (30 mL of glacial acetic acid, 20 mL of phosphoric acid, and 1.25 g of ninhydrin). After heating at 100 °C for 1 h, 4 mL of toluene was added to the solution. The absorbance was measured at 520 nm, and the data were calculated from a standard curve of known concentrations of proline (µmol).

### Principal component analysis (PCA) and pairwise comparisons

The data for starch biosynthesis-related enzymes and starch and amylose contents were analyzed and presented in a biplot of principal component analysis (PCA) using the prcomp software program (Stat version 4.1.0). Pairwise comparison by the Wilcoxon signed rank test was used to compare the data from each group (15 subgroups) with the R package wilcox.test software program (Stat version 4.1.0).

### Statistical analysis

The data were analyzed using a CRD for each trait, and the least significant difference (LSD) test was used to compare the mean differences at an alpha level of 0.05 [35] with the SPSS software program. The data are presented as the means plus standard deviations (SDs) from three independent replicates.

## Results

### Activity of starch biosynthesis-related enzymes in developing grains

**Fig. 1:**
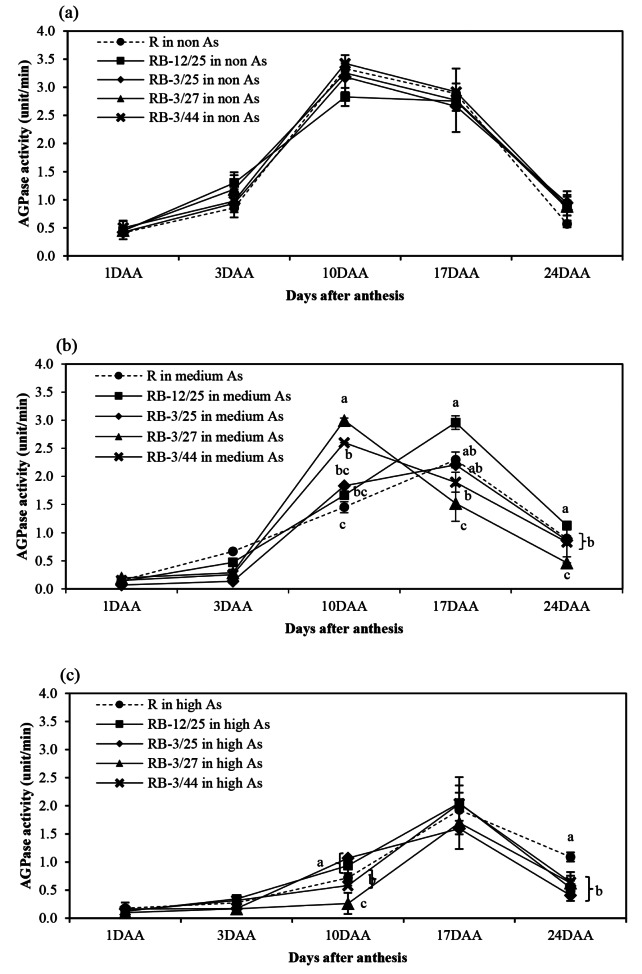
AGPase activity in uninoculated and bacteria-coinoculated rice plants grown during the grain filling period in (**a**) non-As-contaminated soils, (**b**) medium-As-contaminated soils, and (**c**) high-As-contaminated soils. The following treatments were tested: uninoculated rice (R), rice inoculated with KKU2500-12 and 4.25 (RB-12/4.25) bacteria, rice inoculated with KKU2500-3 and 4.25 (RB-3/25) bacteria, rice inoculated with KKU2500-3 and 4.27 (RB-3/4.27) bacteria, and rice inoculated with KKU2500-3 and 4.44 (RB-3/44) bacteria. The values represent the means and standard deviations (*n* = 3). The different letters indicate significant differences among the treatments at each DAA (*p* < 0.05, LSD)

**Fig. 2:**
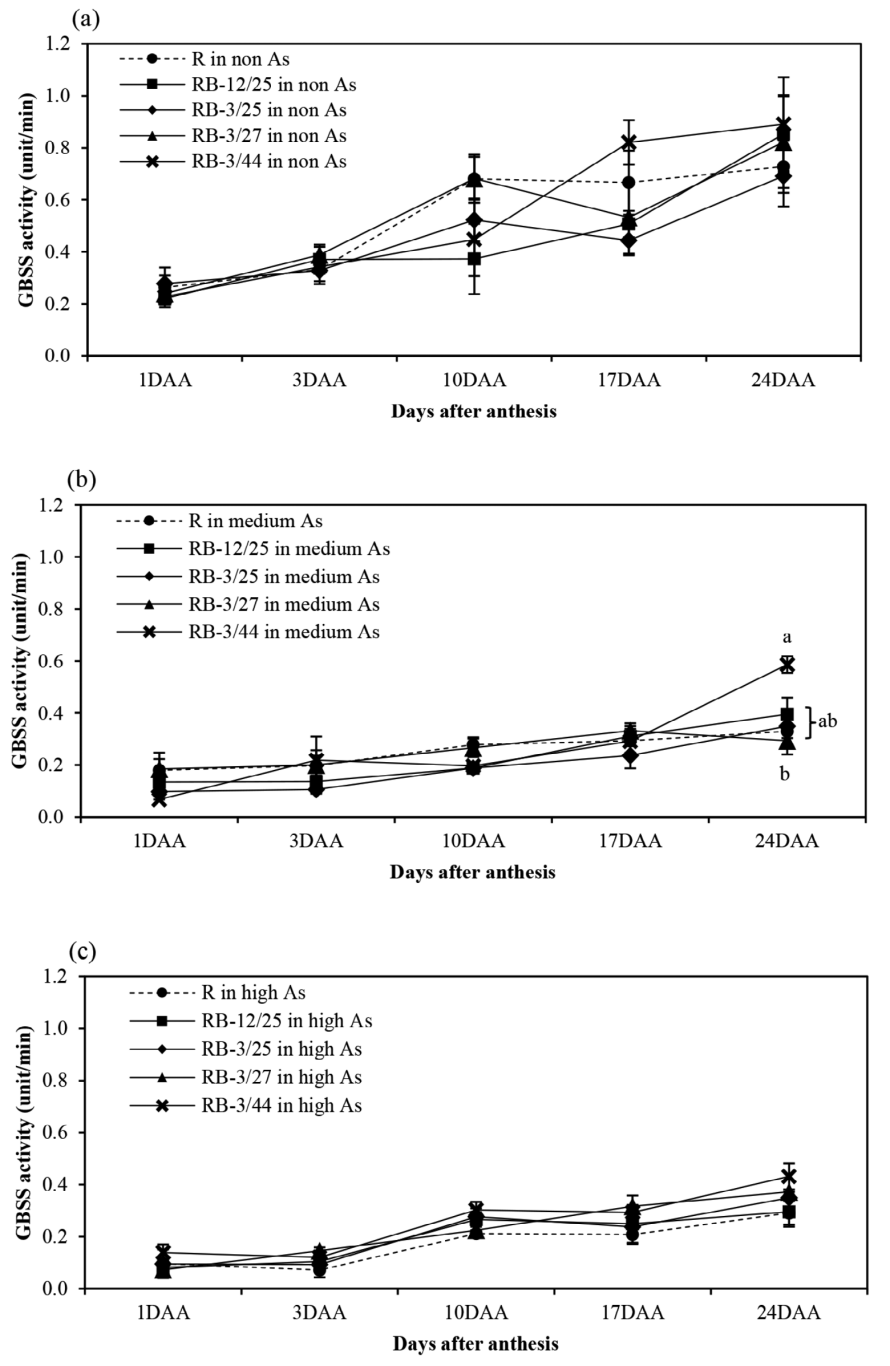
GBSS activity in uninoculated and bacteria-coinoculated rice plants during the grain filling period in (**a**) non-As-contaminated soils, (**b**) medium-As-contaminated soils, and (**c**) high-As-contaminated soils. The following treatments were tested: uninoculated rice (R), rice inoculated with KKU2500-12 and 4.25 (RB-12/4.25) bacteria, rice inoculated with KKU2500-3 and 4.25 (RB-3/25) bacteria, rice inoculated with KKU2500-3 and 4.27 (RB-3/4.27) bacteria, and rice inoculated with KKU2500-3 and 4.44 (RB-3/44) bacteria. The values represent the means and standard deviations (*n* = 3). The different letters indicate significant differences among the treatments at each DAA (*p* < 0.05, LSD)

**Fig. 3:**
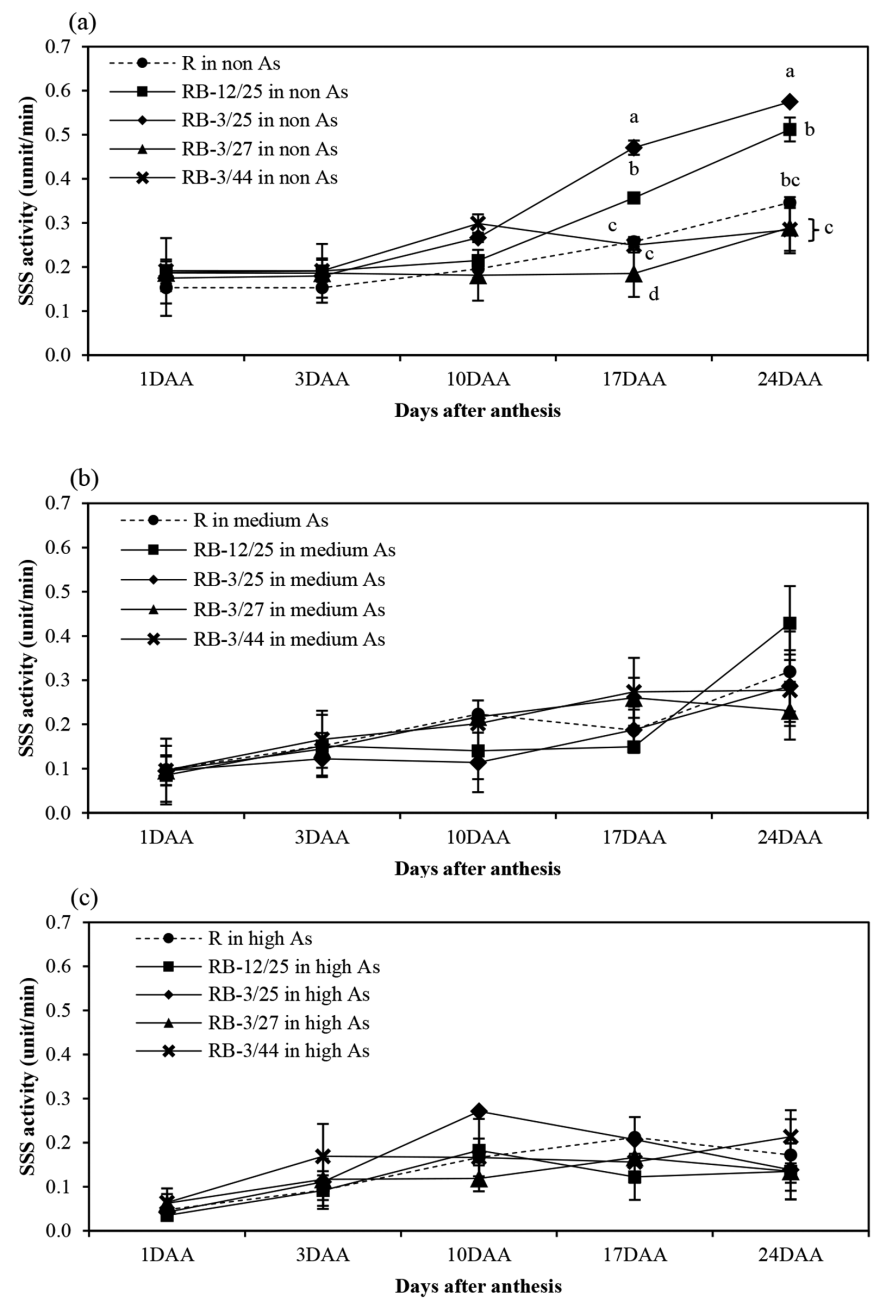
SSS activity in uninoculated and bacteria-coinoculated rice plants grown during the grain filling period in (**a**) non-As-contaminated soils, (**b**) medium-As-contaminated soils, and (**c**) high-As-contaminated soils. The following treatments were tested: uninoculated rice (R), rice inoculated with KKU2500-12 and 4.25 (RB-12/4.25) bacteria, rice inoculated with KKU2500-3 and 4.25 (RB-3/25) bacteria, rice inoculated with KKU2500-3 and 4.27 (RB-3/4.27) bacteria, and rice inoculated with KKU2500-3 and 4.44 (RB-3/44) bacteria. The values represent the means and standard deviations (*n* = 3). The different letters indicate significant differences among the treatments at each DAA (*p* < 0.05, LSD)

**Fig. 4:**
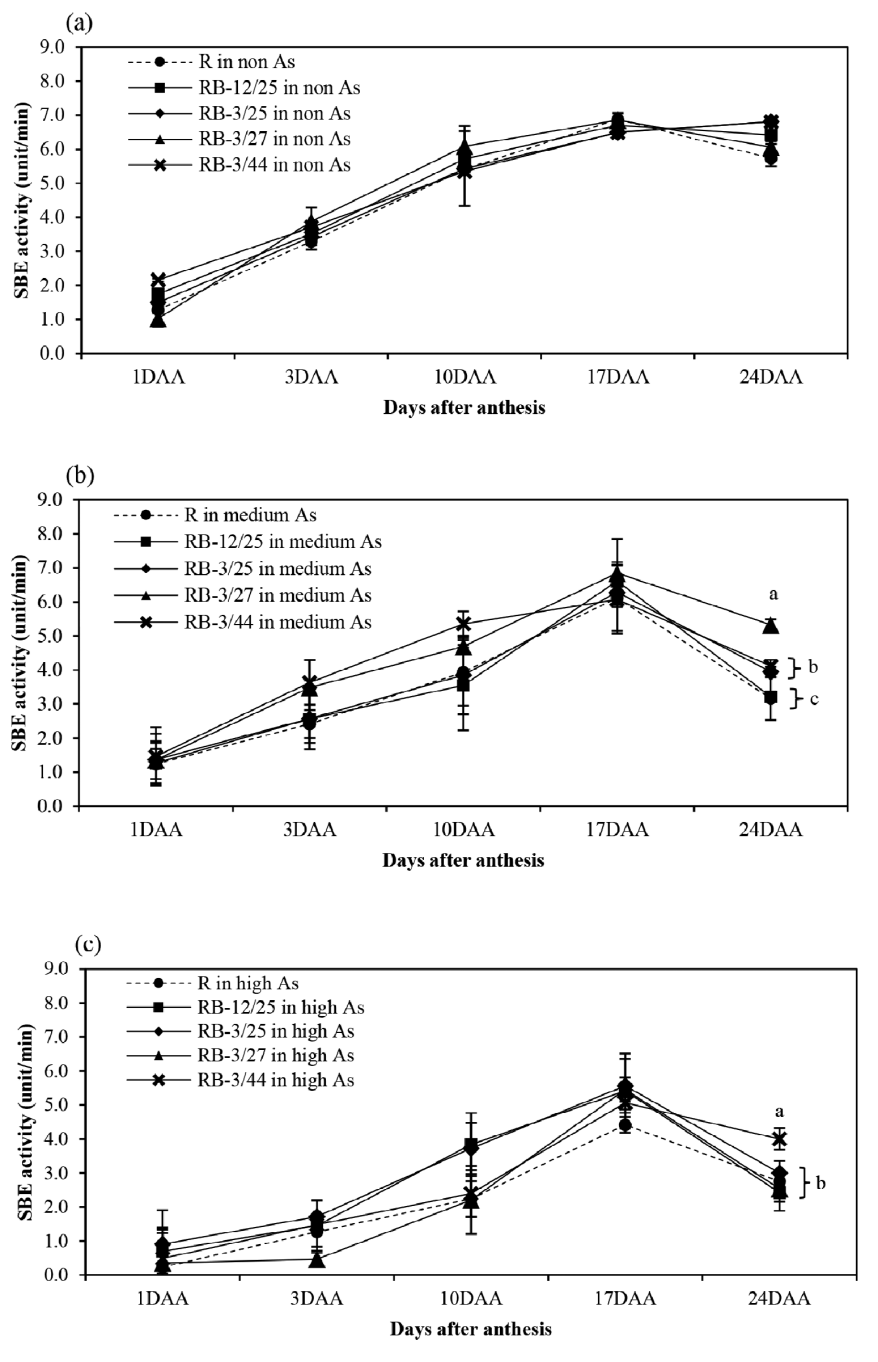
SBE activity in uninoculated and bacteria-coinoculated rice plants grown during the grain filling period in (**a**) non-As-contaminated soils, (**b**) medium-As-contaminated soils, and (**c**) high-As-contaminated soils. The following treatments were tested: uninoculated rice (R), rice inoculated with KKU2500-12 and 4.25 (RB-12/4.25) bacteria, rice inoculated with KKU2500-3 and 4.25 (RB-3/25) bacteria, rice inoculated with KKU2500-3 and 4.27 (RB-3/4.27) bacteria, and rice inoculated with KKU2500-3 and 4.44 (RB-3/44) bacteria. The values represent the means and standard deviations (*n* = 3). The different letters indicate significant differences among the treatments at each DAA (*p* < 0.05, LSD)

**Fig. 5:**
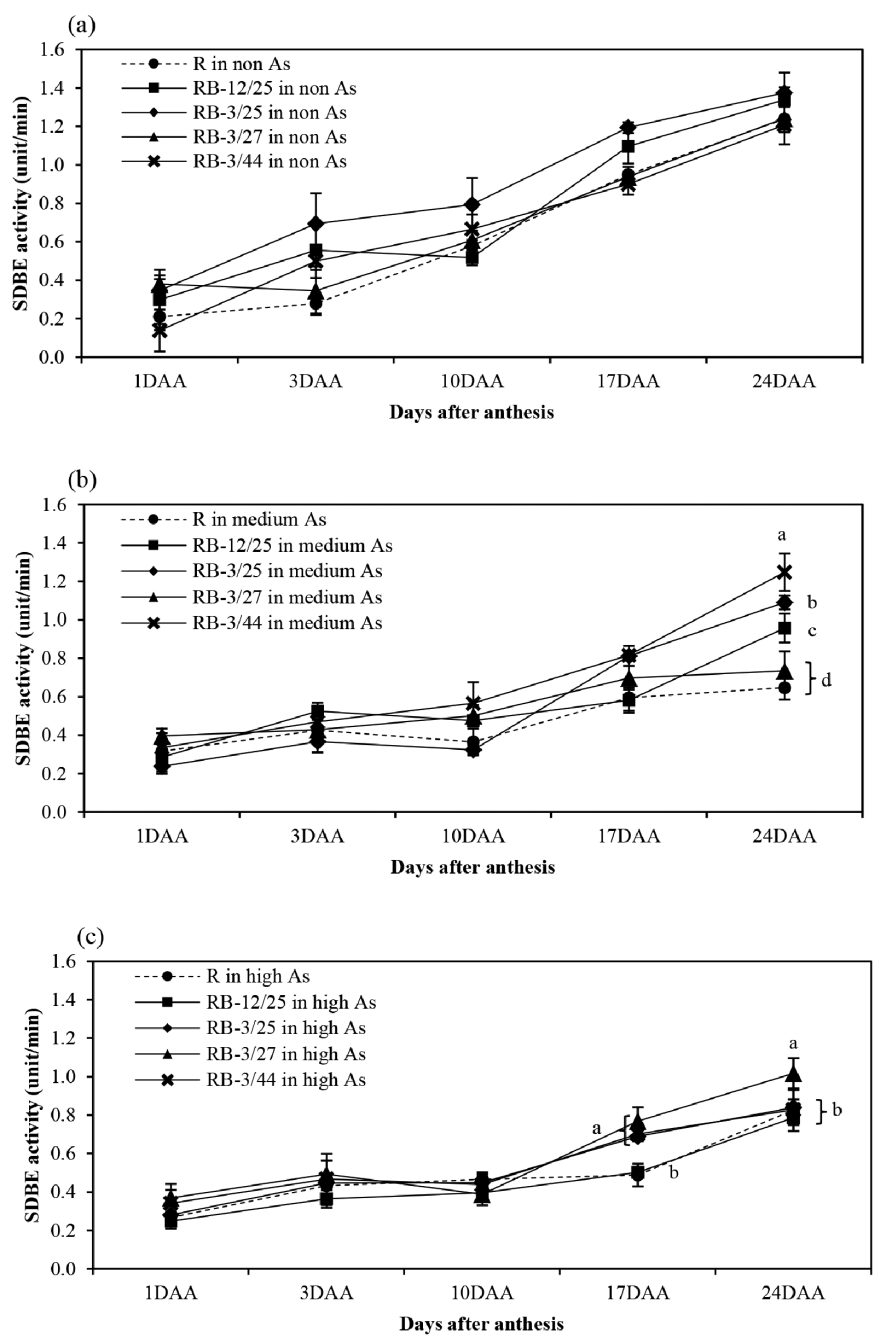
SDBE activity in uninoculated and bacteria-coinoculated rice plants grown during the grain filling period in (**a**) non-As-contaminated soils, (**b**) medium-As-contaminated soils, and (**c**) high-As-contaminated soils. The following treatments were tested: uninoculated rice (R), rice inoculated with KKU2500-12 and 4.25 (RB-12/4.25) bacteria, rice inoculated with KKU2500-3 and 4.25 (RB-3/25) bacteria, rice inoculated with KKU2500-3 and 4.27 (RB-3/4.27) bacteria, and rice inoculated with KKU2500-3 and 4.44 (RB-3/44) bacteria. The values represent the means and standard deviations (*n* = 3). The different letters indicate significant differences among the treatments at each DAA (*p* < 0.05, LSD)

The activity patterns of starch biosynthesis-related enzymes, namely, AGPase, SSS, GBSS, SBE, and SDBE, in rice grown in non-, medium-, and high-As-contaminated soils during grain filling are shown in Figs. 1, 2, 3, 4 and 5. Arsenic affected the activity of all starch biosynthesis-related enzymes. Decreases of 20.25–63.84%, 31.17–59.11%, 5.21–34.07%, 3.06–44.84%, and 3.21–47.71% in the activities of AGPase, GBSS, SSS, SBE, and SDBE, respectively, were detected in rice plants grown in medium-As-contaminated soils. Moreover, an increase in As toxicity was detected in rice plants grown in high-As-contaminated soil. Decreases of up to 33.33–76.27%, 60.04–71.53%, 14.95–49.74%, 35.80-73.39%, and 19.54–47.46% in the activities of AGPase, GBSS, SSS, SBE, and SDBE, respectively, were detected in rice plants grown in high-As-contaminated soils. The activity of these enzymes slightly increased during early grain filling (1–10 DAA). The maximum AGPase activity occurred on day 10 in the non- and medium-As-contaminated soils, whereas the AGPase activity in the high-As-contaminated soil occurred later (17 DAA) (Fig. 1). However, the activity of AGPase decreased after day 17 in all the soils. The SSS, GBSS, and SDBE patterns were similar and exhibited linear increases. GBSS activity decreased when the plants were grown in medium- or high-As-contaminated soils (Fig. 2). Moreover, the GBSS activity did not differ between the medium- and high-As-contaminated soils. The highest activity of SSS occurred on days 17–24 in non-As-contaminated soil. SSS activity occurred later in medium-As-contaminated soil. However, this activity hardly decreased in the high-As-contaminated soil (Fig. 3). The pattern of SBE was similar to that of AGPase, which reflected an increase in early grain filling and a subsequent decrease. The maximal SBE activity occurred on day 17 in all the tested soils (Fig. 4). The SBE activity did not differ between developing grains of rice grown in non- and medium-As-contaminated soils; however, this activity hardly decreased in the high-As-contaminated soil. SDBE activity slightly decreased in the developing grains of rice plants grown in medium- and high-As-contaminated soils (Fig. 5). However, the SDBE activity was lower in high-As-contaminated soil than in medium-As-contaminated soil.

When uninoculated and coinoculated rice were compared, it was found that bacteria enhanced starch biosynthesis-related enzyme activities, which were significantly different. These included AGPase activities at 10–24 DAA and 10 and 24 DAA for developing grains grown in medium- and high-As-contaminated soils, respectively (Fig. 1); GBSS activities at 24 DAA for developing grains grown in medium-As-contaminated soil (Fig. 2); SSS activities at 17–24 DAA for developing grains grown in non-As-contaminated soil (Fig. 3); SBE activities at 24 DAA for developing grains grown in both medium- and high-As-contaminated soils (Fig. 4); and SDBE activities at 24 DAA and 17–24 DAA for developing grains grown in medium- and high-As-contaminated soils, respectively (Fig. 5). Compared with those in uninoculated rice plants, the activities of AGPase, GBSS, SSS, SBE, and SDBE in coinoculated rice grown in As-contaminated soils increased by 1.51–84.37%, 5.52–77.75%, 0.85–32.73%, 1.79–68.58%, and 3.88–68.43%, respectively. In contrast, the enzyme activities of AGPase, GBSS, SSS, SBE, and SDBE in coinoculated rice grown in high-As-contaminated soils were 5.14–43.69%, 0.86–52.33%, 1.27-24.00%, 1.31–42.45%, and 1.59–57.52% greater than those in uninoculated rice, respectively. The combinations of KKU2500-3 and 4.25 (RB-3/25) and of KKU2500-3 and 4.44 (RB-3/44) yielded greater activity than the other combinations of strains.

### Starch accumulation and amylose content in developing grains

**Fig. 6:**
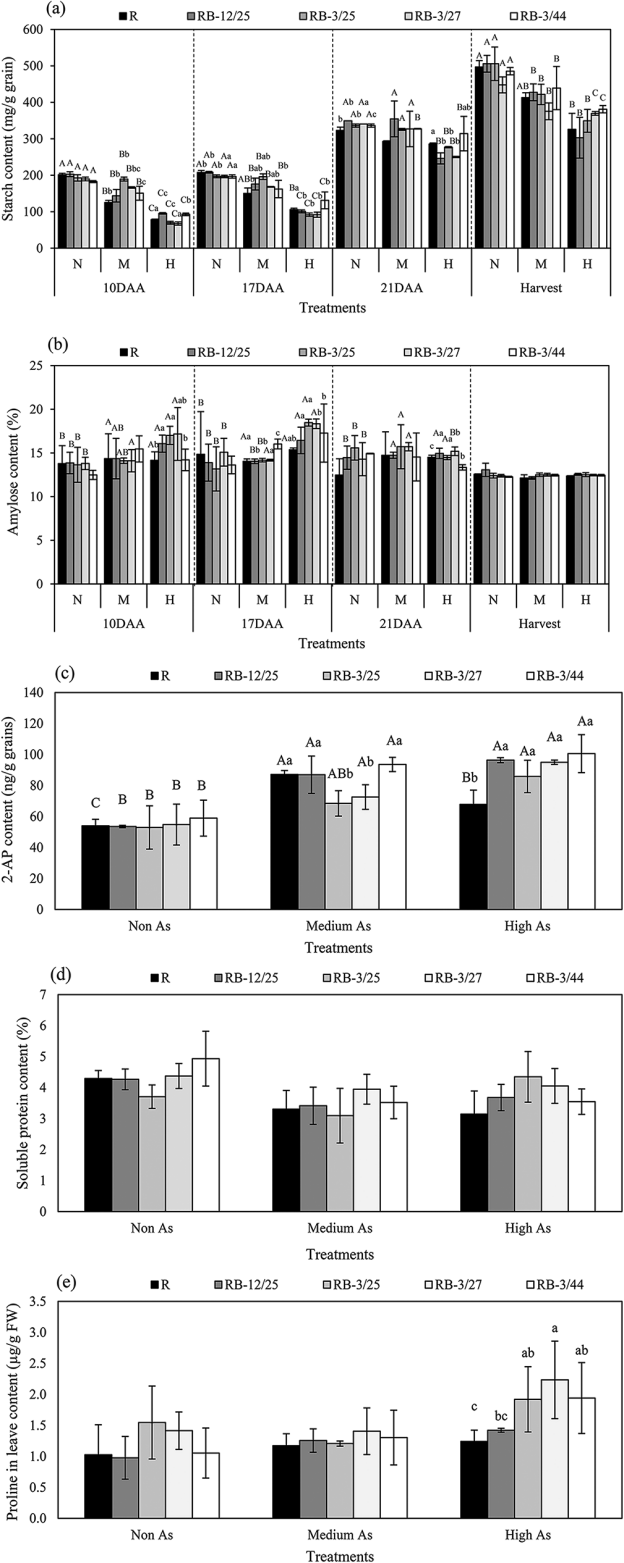
(**a**) Starch content, (**b**) amylose content, (**c**) 2-AP content, (**d**) soluble protein content in the grains, and (**e**) proline content in the leaves of uninoculated and bacteria-coinoculated rice plants grown in non-, medium-, or high-As-contaminated soils. The lowercase letters represent comparisons of the combinations of strains at each As level, and the uppercase letters represent comparisons of As levels in response to each combination of bacterial strains

The starch and amylose contents in developing grains of rice grown in non-, medium- and high-As-contaminated soils are shown in Fig. 6. The accumulation of total starch increased during grain filling (Fig. 6a). However, the contents decreased when the plants were grown in medium- and high-As-contaminated soils. The starch content in rice grown in medium- and high-As-contaminated soils decreased by 9.42–16.91% and 11.57–61.07%, respectively. However, the accumulation of starch in the developing grains of coinoculated plants grown in medium- and high-As-contaminated soils was 2.11–51.16% and 7.11–23.81% greater than that in uninoculated plants, and significant differences were found at 17–24 DAA, 10–17 DAA, and 10–24 DAA in developing plants grown in non-, medium-, and high-As-contaminated soils, respectively. The combinations of KKU2500-3 and 4.25 (RB-3/25) and KKU2500-3 and 4.44 (RB-3/44) resulted in greater starch accumulation than did the other combinations of strains. These strains increased the starch content by up to 51.16% and 23.81% in rice plants grown in medium- and high-As-contaminated soils, respectively, compared with those in uninoculated plants. The content of amylose increased at 10–17 DAA in the plants grown under As conditions but decreased at harvest, but no significant difference in the amylose content, which ranged from 12.14 to 18.47%, was found between soils with different As levels (Fig. 6b). However, the amylose content did not significantly differ between developing grains of uninoculated and coinoculated rice.

### Correlation analysis of starch biosynthesis-related enzymes activities, starch content, and amylose content

**Fig. 7:**
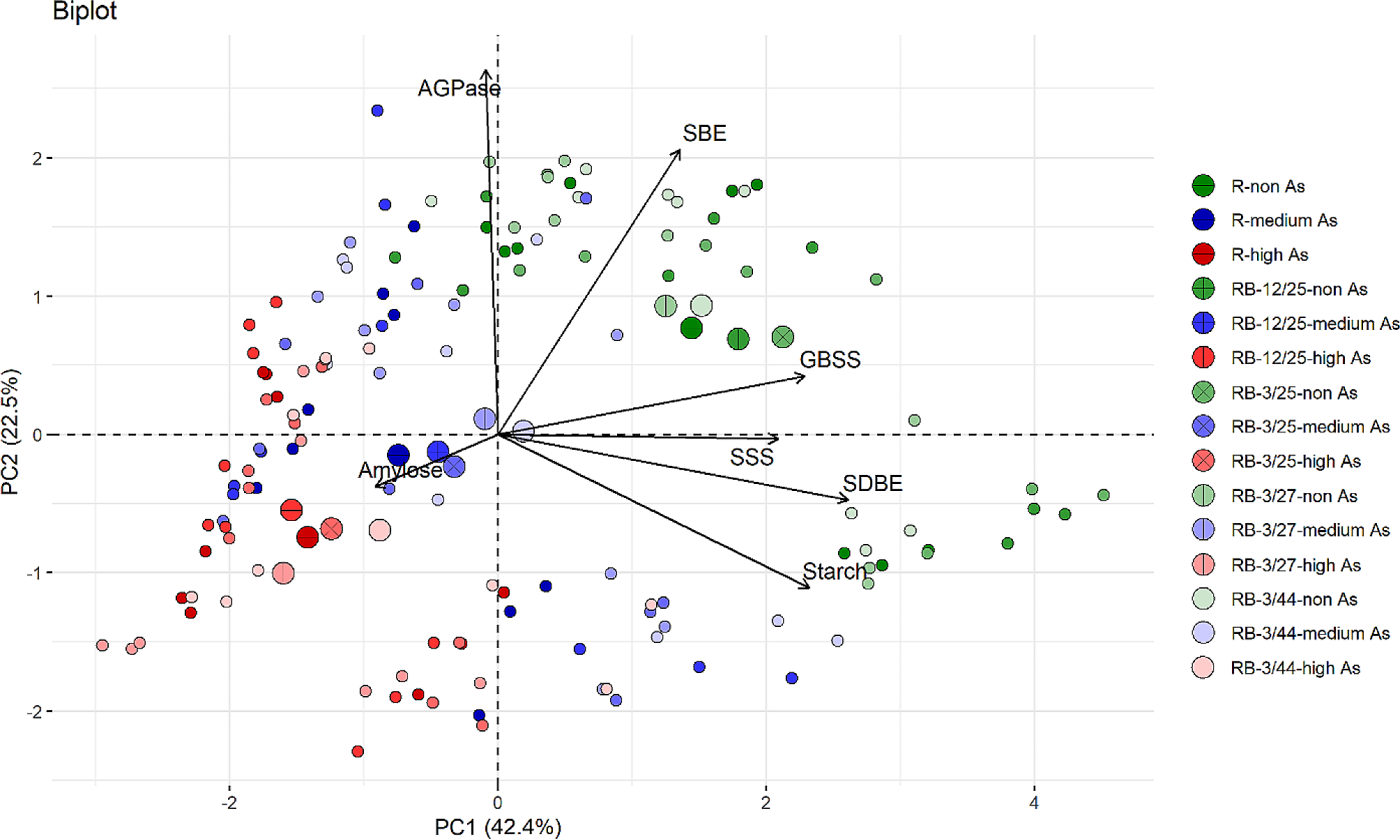
Principal component analysis (PCA) of starch biosynthesis-related enzyme activities, starch content, and amylose content of uninoculated and coinoculated rice plants grown in non-, medium-, or high-As-contaminated soils. The black arrows represent the analyzed variables, and the circles in color and texture represent the combination conditions of bacterial coinoculation and As concentration

The PCA of starch biosynthesis-related enzymes activities, starch content, and amylose content of uninoculated and coinoculated rice plants grown in non-, medium-, and high-As-contaminated soils are shown in Fig. 7. According to the PCA biplot, two principal components (PC1 and PC2) accounted for 64.9% of the total variation. The major groups were divided into 3 groups based on the concentrations of As in the soils (non-, medium-, and high-As), and the subgroups were divided into 15 subgroups based on the bacterial combination treatment and the concentration of As (5 bacterial combination treatments × 3 As levels in the soil). The results of major group analysis demonstrated that increasing SSS, GBSS, SBE, and SDBE enzyme activities and starch content were correlated with rice plant growth in non-As-contaminated soils and PC1, whereas increasing AGPase activity was correlated with rice plant growth in non-As-contaminated soils and PC2. An increase in the amylose content was correlated with rice plant growth in high-As-contaminated soil. The pairwise comparisons by the Wilcoxon signed rank test of 15 subgroups of uninoculated and coinoculated groups are presented in Table 1 and Fig. S1. A significant comparison of enzyme activity was mostly found for GBSS activity, followed by SSS activity, SBE activity, amylose content, and AGPase activity. However, the starch content did not significantly differ among the combined subgroups. In pairwise comparisons of the uninoculated group with the KKU2500-3 and 4.25 (RB-3/25) and KKU2500-3 and 3.44 (RB-3/44) combinations, variations in starch biosynthesis-related enzyme activity were observed but were mostly not significantly different (Table 2 and Fig. S2). In the absence of As, the activities of GBSS and SBE decreased but that of SDBE increased in the RB-3/25 group compared to those in the uninoculated group. However, increases, decreases, and no changes in GBSS, SDBE or SBE activity were observed in RB-3/44. In high-As-contaminated soil, a combination of KKU2500-3 and 3.44 increased GBSS activity in rice plants.

**Table 1:** Pairwise comparison of starch biosynthesis-related enzyme activities, starch content, and amylose content using the Wilcoxon signed rank test of 15 subgroups based on bacterial combination treatment and the concentration of As (5 combination treatments x 3 As levels in soils)

Treatment	Pairwise comparison	Starch biosynthesis-related enzyme activity										Starch content		Amylose content	
		AGPase		GBSS		SSS		SBE		SDBE					
		p value	sig	p value	sig	p value	sig	p value	sig	p value	sig	p value	sig	p value	sig
R	Non As vs. Medium As	0.34	ns	4.10E-05	****	0.6	ns	0.024	*	0.019	*	0.05	ns	0.66	ns
	Non As vs. High As	0.26	ns	4.10E-05	****	0.05	ns	0.00016	***	0.004	**	0.05	ns	0.22	ns
	Medium As vs. High As	0.39	ns	0.031	*	0.024	*	0.094	ns	0.73	ns	0.05	ns	0.33	ns
RB-12/25	Non As vs. Medium As	1	ns	0.014	*	0.031	*	0.031	*	0.052	ns	0.16	ns	0.0056	**
	Non As vs. High As	0.04	*	0.004	**	8.20E-05	****	0.0028	**	0.017	*	0.052	ns	0.034	*
	Medium As vs. High As	0.05	ns	0.57	ns	0.13	ns	0.86	ns	0.22	ns	0.05	ns	0.48	ns
RB-3/25	Non As vs. Medium As	0.077	ns	0.00041	***	0.0028	**	0.94	ns	0.04	*	0.55	ns	0.67	ns
	Non As vs. High As	0.034	*	0.00029	***	0.0027	**	0.0012	**	0.004	**	0.05	ns	0.031	*
	Medium As vs. High As	0.077	ns	0.63	ns	0.96	ns	0.67	ns	0.55	ns	0.05	ns	0.014	*
RB-3/27	Non As vs. Medium As	0.19	ns	8.20E-05	****	0.19	ns	0.34	ns	0.077	ns	0.11	ns	0.35	ns
	Non As vs. High As	0.004	**	4.10E-05	****	0.042	*	0.0019	**	0.063	ns	0.052	ns	0.014	*
	Medium As vs. High As	0.22	ns	0.93	ns	0.00041	***	0.011	*	0.39	ns	0.05	ns	0.19	ns
RB-3/44	Non As vs. Medium As	0.11	ns	0.0056	**	0.44	ns	0.11	ns	0.73	ns	0.14	ns	0.031	*
	Non As vs. High As	0.004	**	0.004	**	0.0012	**	0.0012	**	0.042	*	0.14	ns	0.22	ns
	Medium As vs. High As	0.077	ns	0.8	ns	0.004	**	0.14	ns	0.16	ns	0.19	ns	0.73	ns

Statistically significant (sig) p values: **** <0.0001, *** <0.001, ** <0.01, * <0.05, ns > 0.05

**Table 2:** Pairwise comparison of starch biosynthesis-related enzyme activities, starch content, and amylose content using the Wilcoxon signed rank test of uninoculated (R) and rice plants coinoculated with the RB-3/25 and RB-3/44 bacterial combinations under non-, medium- and high-As concentrations

Treatment	Pairwise comparison	Starch biosynthesis-related enzyme activity										Starch content		Amylose content	
		AGPase		GBSS		SSS		SBE		SDBE					
		p value	sig	p value	sig	p value	sig	p value	sig	p value	sig	p value	sig	p value	sig
R vs. RB-3/25	R-Non As vs. RB-3/25 Non As	0.93	ns	0.094	ns	0.063	ns	0.49	ns	0.14	ns	0.6	ns	0.67	ns
	R-Non As vs. RB-3/25 Medium As	0.26	ns	0.00041	***	0.12	ns	0.11	ns	0.39	ns	0.23	ns	0.6	ns
	R-Non As vs. RB-3/25 High As	0.052	ns	0.000082	****	0.093	ns	0.0056	**	0.05	ns	0.05	ns	0.014	*
	R-Medium As vs. RB-3/25 Medium As	0.73	ns	0.45	ns	0.19	ns	0.8	ns	0.26	ns	0.05	ns	0.43	ns
	R-Medium As vs. RB-3/25 High As	0.18	ns	0.4	ns	0.11	ns	0.8	ns	0.05	ns	0.05	ns	0.017	*
	R-High As vs. RB-3/25 High As	0.38	ns	0.17	ns	0.63	ns	0.16	ns	0.8	ns	0.26	ns	0.077	ns
R vs. RB-3/44	R-Non As vs. RB-3/44 Non As	0.39	ns	0.26	ns	0.89	ns	0.6	ns	0.86	ns	0.63	ns	0.67	ns
	R-Non As vs. RB-3/44 Medium As	0.34	ns	0.0012	**	0.86	ns	0.16	ns	0.73	ns	0.14	ns	0.094	ns
	R-Non As vs. RB-3/44 High As	0.094	ns	0.00016	***	0.1	ns	0.0012	**	0.052	ns	0.16	ns	0.22	ns
	R-Medium As vs. RB-3/44 Medium As	0.11	ns	0.31	ns	0.034	*	0.51	ns	0.11	ns	0.44	ns	0.66	ns
	R-Medium As vs. RB-3/44 High As	0.73	ns	0.93	ns	1	ns	0.49	ns	0.05	ns	0.17	ns	0.29	ns
	R-High As vs. RB-3/44 High As	0.3	ns	0.024	*	0.93	ns	0.34	ns	0.93	ns	0.44	ns	0.89	ns

Statistically significant (sig) p values: **** <0.0001, *** <0.001, ** <0.01, * <0.05, ns > 0.05

### Aromatic compound and soluble protein contents in the grains

The 2-AP contents in the grains are shown in Fig. 6c. The 2-AP content significantly increased in the rice plants grown in As-contaminated soil, and increases in the 2-AP content were found with increasing soil As content in all the seedlings, except uninoculated rice. Increases in the 2-AP content of 61.30% and 25.58% were detected in rice plants grown in medium- and high-As-contaminated soils, respectively. Moreover, the bacterium-induced increase in 2-AP content was greater in inoculated rice than in uninoculated rice, and the only exception was found in rice grown in medium-As-contaminated soil and treated with RB-3/25 and RB-3/27. Similarly, compared with those in uninoculated plants, the 2-AP contents in the grains of coinoculated plants were 2.16–9.93% and 26.57–42.04% greater in rice plants grown in medium- and high-As-contaminated soils, respectively.

The soluble protein contents in the grains are shown in Fig. 6d; the contents were not significantly different among the As-contaminated soils or among the different bacterial strain combinations. Thus, As did not affect the soluble protein content in the grains. However, the soluble protein content decreased in As-contaminated soil and was in the range of 8.08–27.95% and 5.59–26.70% in the medium- and high-As-contaminated soils, respectively.

The proline contents in the leaves are shown in Fig. 6e; the proline contents increased significantly by 14.19–20.95% in the rice plants grown in As-contaminated soil. Moreover, the bacterium-induced increase in proline content was greater in the inoculated rice than in the uninoculated rice, particularly in those grown in soil contaminated with medium levels of As. Similarly, the coinoculation of rice plants in medium- and high-As-contaminated soils increased the proline content in the leaves by 2.96–19.95% and 14.53–80.04%, respectively. In addition, significant differences in the proline content of seedlings grown in high-As-contaminated soil were found among the different bacterial strain combinations.

## Discussion

Arsenic is known to be phytotoxic to rice growth and grain production. Toxicity reduces various growth parameters and the productivity of KDML105 rice grown in As-contaminated soil. With respect to rice productivity, the filled-grain percentage was hardly reduced under As stress, particularly due to high-As-contaminated soil, leading to a decrease in grain weight [11]. This research indicated that As toxicity reduced the activity of starch biosynthesis-related enzymes related to the accumulation of starch in the grains (Figs. 1, 2, 3, 4, 5 and 6). The key enzymes involved in starch biosynthesis include AGPase, SSS, GBSS, SBE, and SDBE [13, 36]. AGPase is involved in the first major regulatory step in starch biosynthesis; this enzyme produces ADP-glucose, which is a precursor to starch [37]. Thus, a reduction in AGPase activity leads to a reduction in the starch content during grain filling. The activity of AGPase decreased when rice was grown in As-contaminated soil (Fig. 1), and this change occurred later than when rice was grown in non-As-contaminated soil; this resulted in a direct reduction in starch accumulation in rice grown in As-contaminated soil. A variety of starch synthases, including SSS, GBSS, SBE, and SDBE, can be manipulated to change the starch quality and quantity, which affects the amylose and amylopectin contents [13]. Many starch synthases exist in different isoforms, and they all play a role in elongating linear glucan chains and synthesizing amylose or other chain subclasses of amylopectin [37]. Amylose is synthesized by AGPase and GBSS, while amylopectin is synthesized through linkage-type reactions by AGPase, SSS, SBE, and SDBE [27, 38]. Thus, relatively low GBSS activity is continually supported by a decreasing amylose content in starch reserves [13]. The present study showed that the activity of SSS and GBSS was affected by As toxicity, as rice grown in As-contaminated soil had lower activity than rice grown in noncontaminated soil (Figs. 2 and 3).

According to the PCA results, the overall reduction in the activity of starch biosynthesis-related enzymes and starch content of both uninoculated and coinoculated plants were correlated with their growth in As-contaminated soils (Fig. 7). Pairwise comparisons between two individual groups revealed that the p values of GBSS activity were significantly different between all 3 levels of As in the uninoculated group (Table 1). In contrast, for each bacterial combination, significant differences in GBSS activity were detected between non-As-treated plants and medium- and high-As-treated plants but not between plants grown in medium- and high-As-treated plants. Based on these results, bacteria may be effective at reducing As toxicity in the range of 29.91 mg/kg (medium) to 45.11 mg/kg (high) As in soil without affecting GBSS activity (Table 1). The amylose content increased in rice plants grown in As-contaminated soil during the early grain filling period (Fig. 6b), which was consistent with the PCA results (Fig. 7). These results might be attributed to the decreases in SBE and SDBE in rice grown in As-contaminated soil (Figs. 4 and 5). SBE plays a crucial role in the branching of amylopectin during its biosynthesis; SBE is the only enzyme that links α-1,6-glycosides to α-polyglucans [39]. A pairwise comparison revealed that the activity of some enzymes varied depending on the enzyme type, bacterial combination strains and level of As in the soil (Tables 1 and 2). Toxicity significantly reduced SBE and SDBE activities in soil contaminated with As in the uninoculated group (Table 1 and Fig. S1). However, inoculation with KKU2500-3 and 4.25 (RB-3/25) or KKU2500-3 and 4.44 (RB-3/44) had no effect on SBE activity in rice plants grown in soils with non As or medium As. Furthermore, RB-3/25 and RB-3/44 maintained the SBE and SDBE activities of the plants to the same level when grown in media with high As concentrations (Table 2 and Fig. S2). Interestingly, RB-3/44 exhibited significantly greater GBSS activity than uninoculated plants in high-As-contaminated soil. These results indicated that As was directly toxic to several starch biosynthesis-related enzymes in rice plants. The bacterial strains and combination affect these enzyme activities in different manners and under different levels of As and depending on the postanthesis period. However, it should be noted that starch biosynthesis in rice plants is complex and involves several plant enzymes. To understand the true mechanisms related to the effects of As and/or bacteria on starch biosynthesis, particular bacterial strains and combinations and activities of certain key enzymes should be investigated further comprehensively.

Microbial bioremediation can be used to mitigate the toxicity of environmental pollution via microorganism activity. The As(III)-oxidizing bacteria *P*. *stutzeri* 4.25, 4.27, and 4.44 and the plant growth-promoting bacteria *D*. *acidovorans* KKU2500-12 and *C*. *taiwanensis* KKU2500-3 used in the present study were isolated from heavy metal-contaminated areas in Thailand [24, 25]. According to our previous studies by Thongnok et al. (2018) [25], there was a substantial decrease in As toxicity in rice under closed-system hydroponic conditions when PGPB and As(III)-oxidizing bacteria were combined in four combinations: *D. acidovorans* KKU2500-12 and *P. stutzeri* 4.25 (RB-12/25) and *C. taiwanensis* KKU2500-3 and *P. stutzeri* strains 4.25 (RB-3/25), 4.27 (RB-3/27), and 4.44 (RB-3/44). According to Thongnok et al. (2021) [11], in nutrient media supplemented with As(III), *P. stutzeri* strains 4.25, 4.27, and 4.44 were proven to be As(III)-oxidizing bacteria capable of converting highly toxic As(III) to As(V) and immobilizing As in soil and plants during bioremediation by utilizing direct and indirect mechanisms to increase plant growth under stress. This study determined that the combination of RB-12/25, RB-3/25, RB-3/27, or RB-3/44 reduced the accumulation of As in rice grains by 24–100% when the soil contained 29.13 mg/kg As, which is lower than the recommendation of Codex Alimentarius (2019) [40] for 0.35 mg/kg husked rice. Furthermore, As in rice grains was also reduced by 17–50% with these four bacterial combinations in soil at 45.11 mg/kg As. The results of this study indicate that the abovementioned bacterial combinations significantly reduce the accumulation of As in rice grains under greenhouse conditions. Additionally, the same combinations reduced As toxicity and improved rice growth and productivity under As conditions. Furthermore, they increase grain yields by reducing the percentage of unfilled grains. In a recent study, the same bacterial combinations were also found to be able to reduce *V*_*max*_ values and increase *K*_*m*_ values in an As uptake and transport kinetic assay [41].

The results of this present study revealed that the combination of bacteria increased starch biosynthesis-related enzyme activity in developing grains, resulting in an increase in starch accumulation in those grains (Figs. 1, 2, 3, 4, 5, 6 and 7; Tables 1 and 2). As(III) can react with the -SH groups of enzymes, inhibiting enzyme activity and cellular functions in plants [6]. It is likely that the As(III) in inoculated rice can be converted to As(V) through oxidation by bacteria; therefore, reducing As(III) may enhance the activity of enzymes involved in starch biosynthesis. Moreover, the ability to enhance plant growth by plant growth-promoting bacteria is also important and is directly related to the grain filling process. The grain filling ratio can be determined on the basis of the complex sink–source balance in plants [42, 43]. Thus, the reduction in the effects of As on plant growth and development positively affects carbohydrate supplies (source ability), resulting in an increase in the starch biosynthesis process (sink strength).

The starch, amylose and amylopectin contents are indicators of grain quality, and volatile compounds in grains are also important because of their pleasant taste and smell, resulting in fragrance, which is desired by the global population. More than 200 volatile compounds have been detected in rice, and 2-AP is mainly responsible for the specific aroma of rice [44, 45]. Many studies have shown that the accumulation of 2-AP is promoted under stress conditions, especially under salt and drought conditions. In fact, the precursor of 2-AP is proline [16, 46], which is induced in plants under stress related to redox state balance. Thai jasmine rice, or KDML105, is fragrant rice and the most important cultivar in Thailand; this cultivar is widely reported to have the strongest grain aroma. In the present study, the 2-AP content in the grains significantly increased when the plants were grown in As-contaminated soil (Fig. 6c). As a well-known inducer of reactive oxygen species (ROS), proline is induced to protect against ROS-induced damage by As in plants [47]. This phenomenon in rice plants might be affected by As, resulting in the accumulation of proline and an increase in 2-AP in grains. Moreover, the accumulation of proline may also be induced by bacteria. This mechanism is responsible for reducing bacterial stress in plants. The proline content in the leaves hardly increased in the inoculated seedlings grown in the soil with a medium As concentration (Fig. 6e). Thus, this study revealed that the bacteria promoted an increase in the 2-AP content in the grains, and this content was greater in the inoculated rice than in the uninoculated rice. Soluble proteins, which constitute important components of rice, are the second most abundant components in grain, and these proteins directly impact rice quality; specifically, soluble proteins are associated with reduced grain breakage during the milling process [48]. In the present study, As did not affect the soluble protein content in the grains (Fig. 6d).

**Fig. 8:**
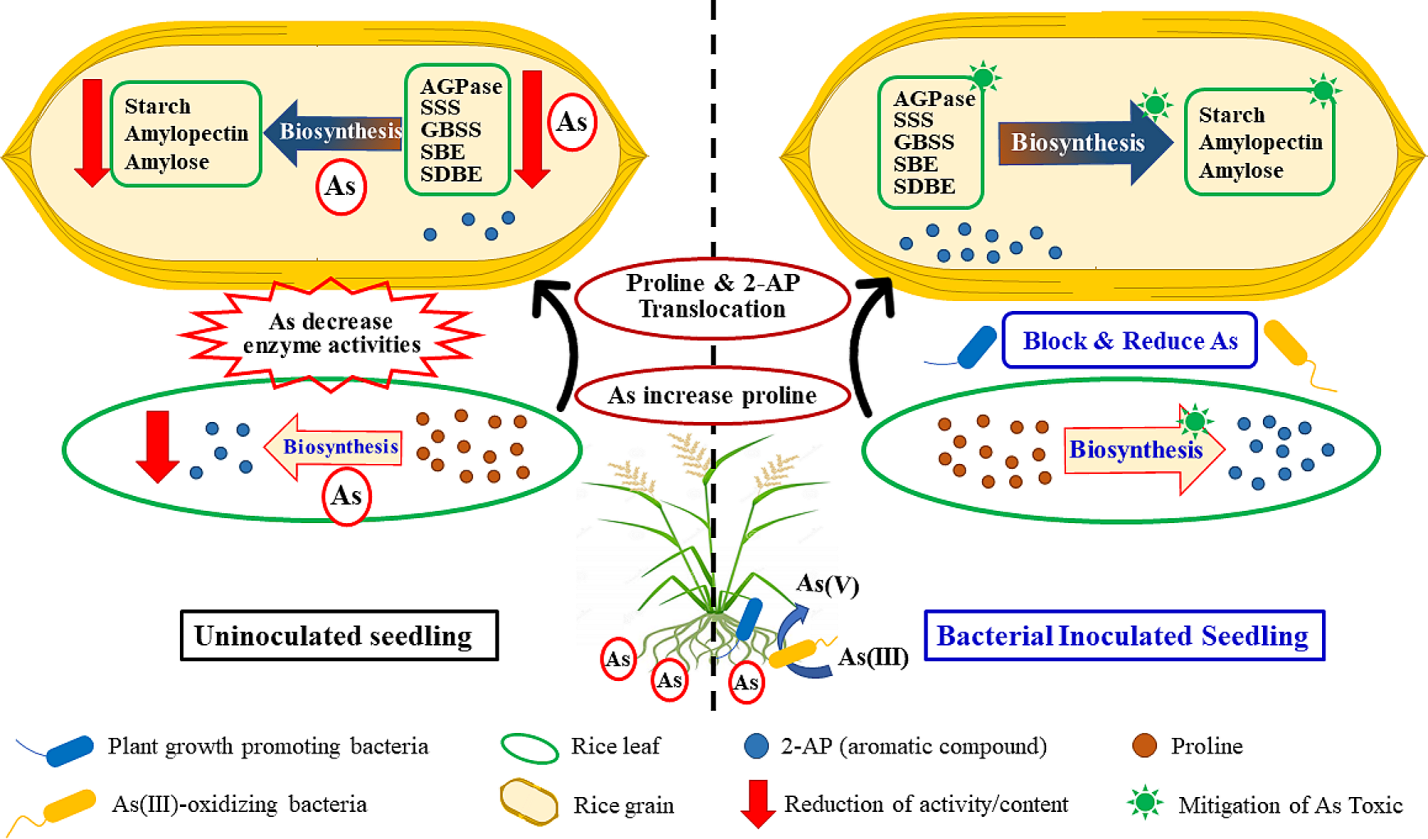
Summary of the mechanisms and bacteria-As-rice plant interactions of starch biosynthesis-related enzymes involved in starch accumulation. Starch accumulation during grain filling is affected by decreasing the activity of starch biosynthesis-related enzymes. Bacteria reduce the effects of As toxicity on rice. As(III)-oxidizing bacteria oxidize highly toxic As(III) to the less toxic As(V), resulting in a decrease in the effect of As toxicity on rice plants, while plant growth-promoting bacteria enhance rice growth. The combinations of bacteria were able to maintain enzyme activities related to increasing starch accumulation. As induced proline accumulation along with an increase in 2-AP content in the grains. AGPase: ADP-glucose pyrophosphorylase; SSS: soluble starch synthase; GBSS: granule-bound starch synthase; SBE: starch-branching enzyme; SDBE: starch-debranching enzyme

The proposed mechanisms of bacteria-As-plant interactions on starch accumulation are summarized in Fig. 8. Starch biosynthesis-related enzymes were affected by As toxicity. This was related to decreased starch accumulation in the grains, which resulted in low rice grain yield and quality. Interestingly, the combination of As(III)-oxidizing bacteria with plant growth-promoting bacteria maintained enzyme activities related to starch accumulation in rice. These findings are important and could help clarify yield and yield component reductions in rice in the future. These findings could also be expanded to improve the production of quality rice grains in As-contaminated areas.

## Conclusion

Arsenic negatively affected the activity of starch biosynthesis-related enzymes, namely, AGPase, SSS, GBSS, SBE, and SDBE, which resulted in decreased starch accumulation and amylose content during grain filling. The combination of As(III)-oxidizing and plant growth-promoting bacteria enhanced starch biosynthesis-related enzyme activities in rice cultivated in soils contaminated with different As concentrations. Combinations of KKU2500-3 with 4.25 and 4.44 resulted in high enzyme activity, resulting in increased starch accumulation in the grains and high grain yield. Moreover, bacteria and/or As enhanced the 2-AP aromatic compound content in the grains and thus improved rice grain quality. These findings demonstrate that bacteria potentially and functionally mitigate As toxicity by helping maintain starch biosynthesis and amylose accumulation to improve grain yield and quality, and it increase the 2-AP content to improve the grain quality under greenhouse conditions.

### Electronic supplementary material

Below is the link to the electronic supplementary material.

Supplementary Material 1

## Data Availability

All data generated or analyzed during this study are included in this published article. We hereby declare that the data used in this study is included in Supplementary Information (Figures S1 and S2 and Table S1) and have the right to use them. The datasets generated and/or analyzed during the current study are available from the corresponding author upon reasonable request.

## Abbreviations

AGPase: ADP-glucose pyrophosphorylase
DAA: days after anthesis
DAT: days after transplanting
GBSS: granule-bound starch synthase
KDML105: Khao Dawk Mali 105 rice
R: uninoculated rice
RB: bacterium-inoculated rice
SBE: starch-branching enzyme
SDBE: starch-debranching enzyme
SSS: soluble starch synthase
