# Supplementary material for: As(III)-oxidizing and plant growth-promoting bacteria increase the starch biosynthesis-related enzyme activity, 2-AP levels, and grain quality of arsenic-stressed rice plants
Source: BMC Plant Biol. 2024 Jul 15;24:672. doi: 10.1186/s12870-024-05352-6 (PMC11247763; doi:10.1186/s12870-024-05352-6)
Supplement: Supplementary file 1 — Supplementary Material 1 [file 12870_2024_5352_MOESM1_ESM.docx]

**Supplementary data**

| 1. AGPase activity |
| --- |
| 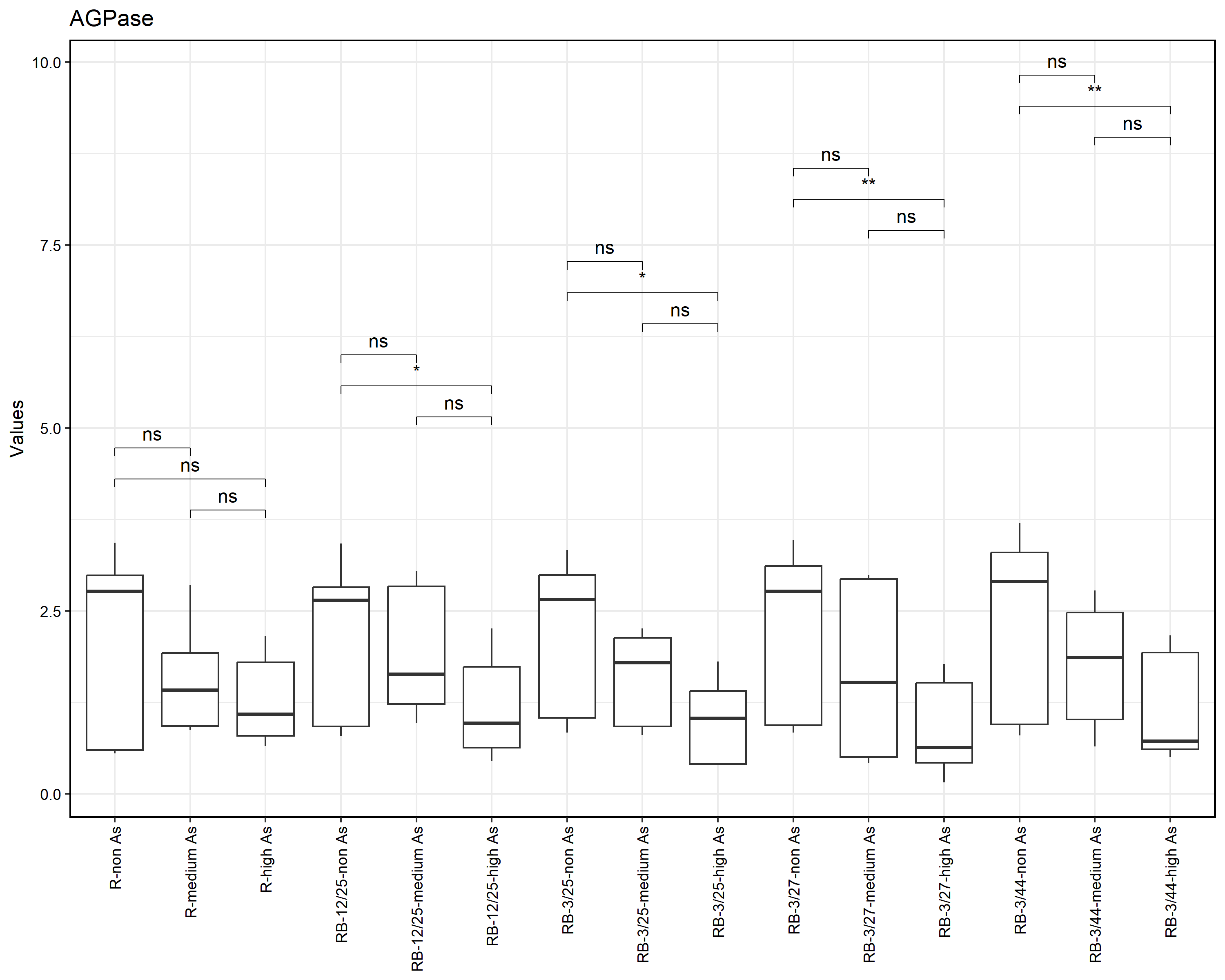 |
| 1. GBSS activity |
| 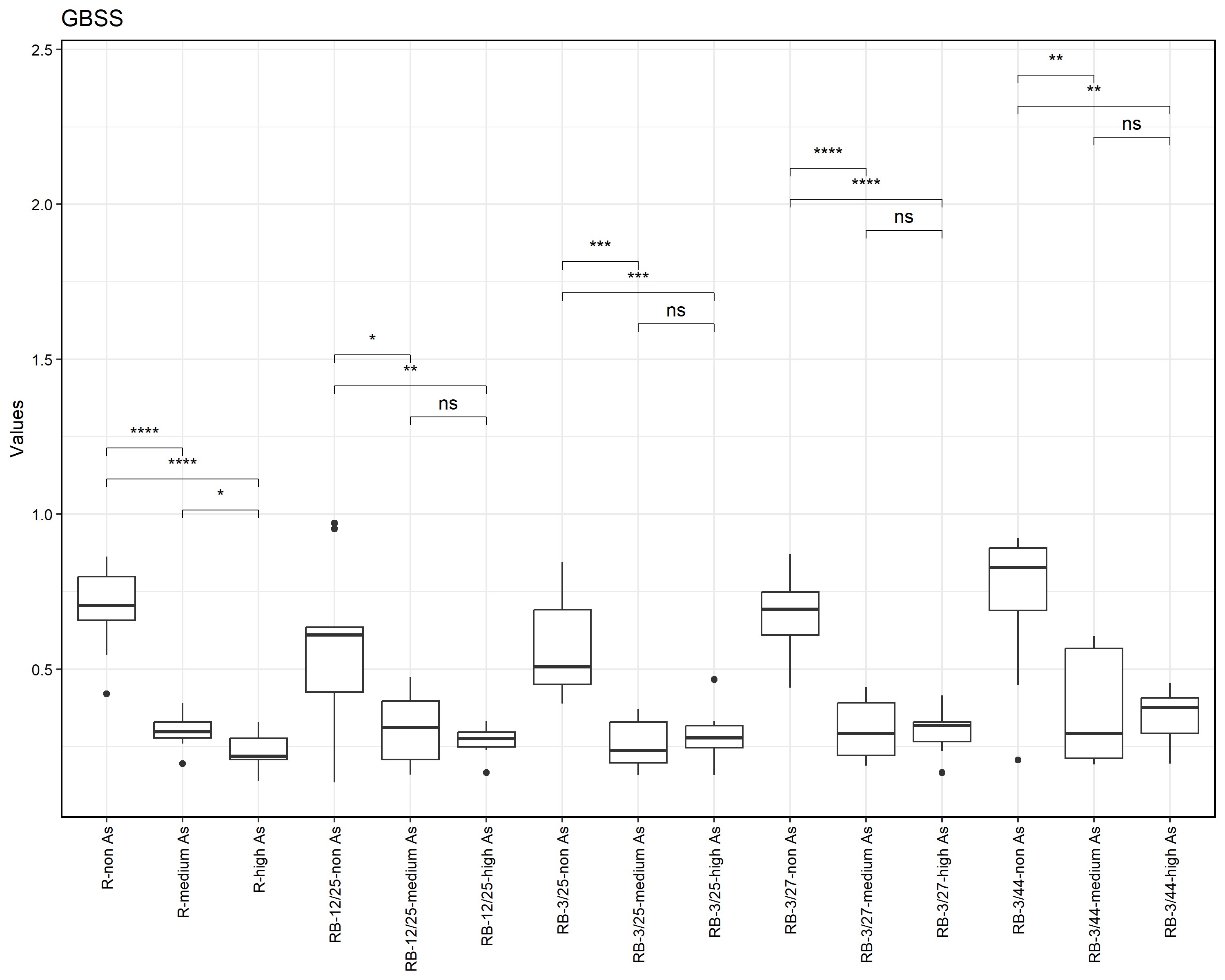 |
|  |
| 1. SSS activity |
| 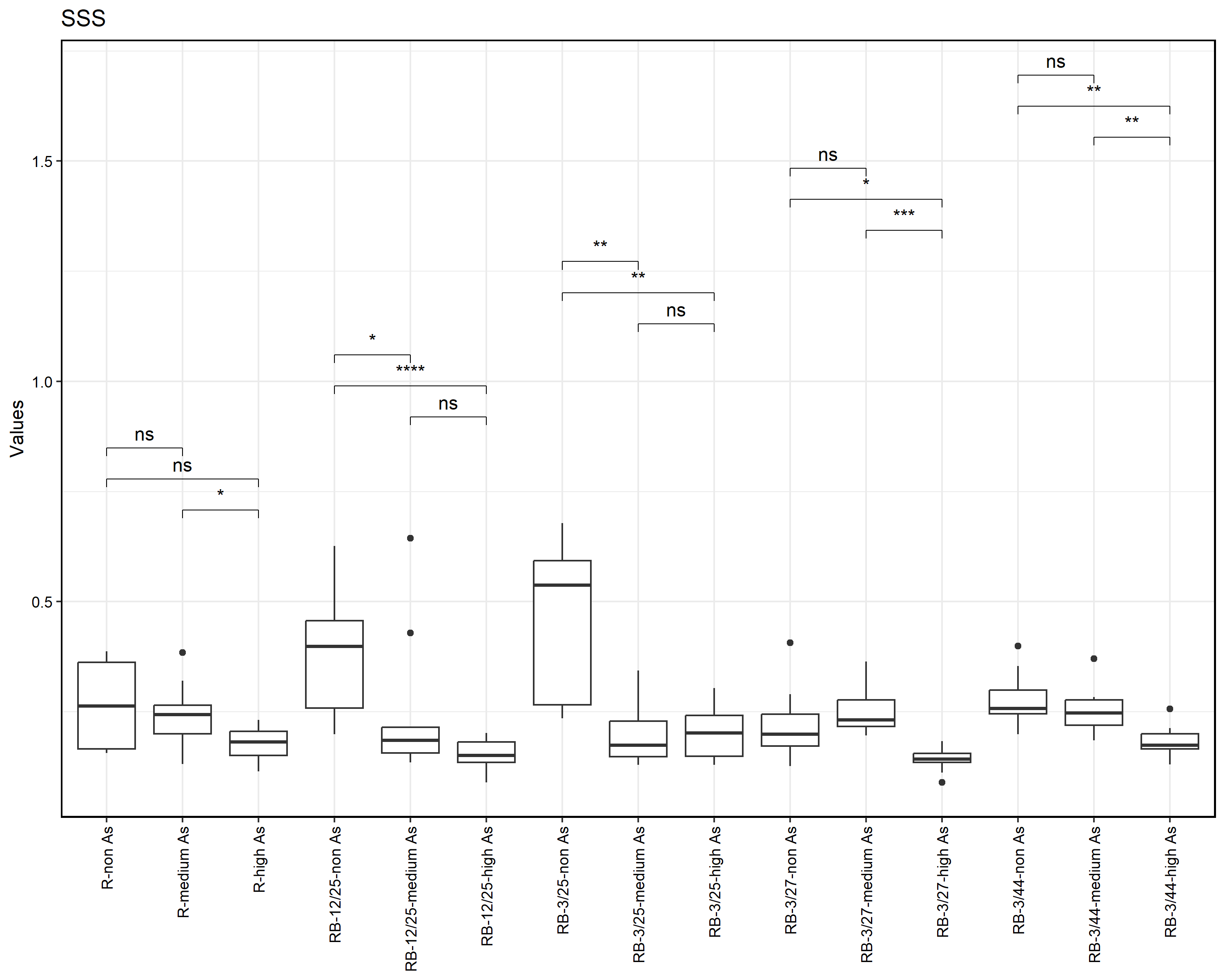 |
|  |
| 1. SBE activity |
| 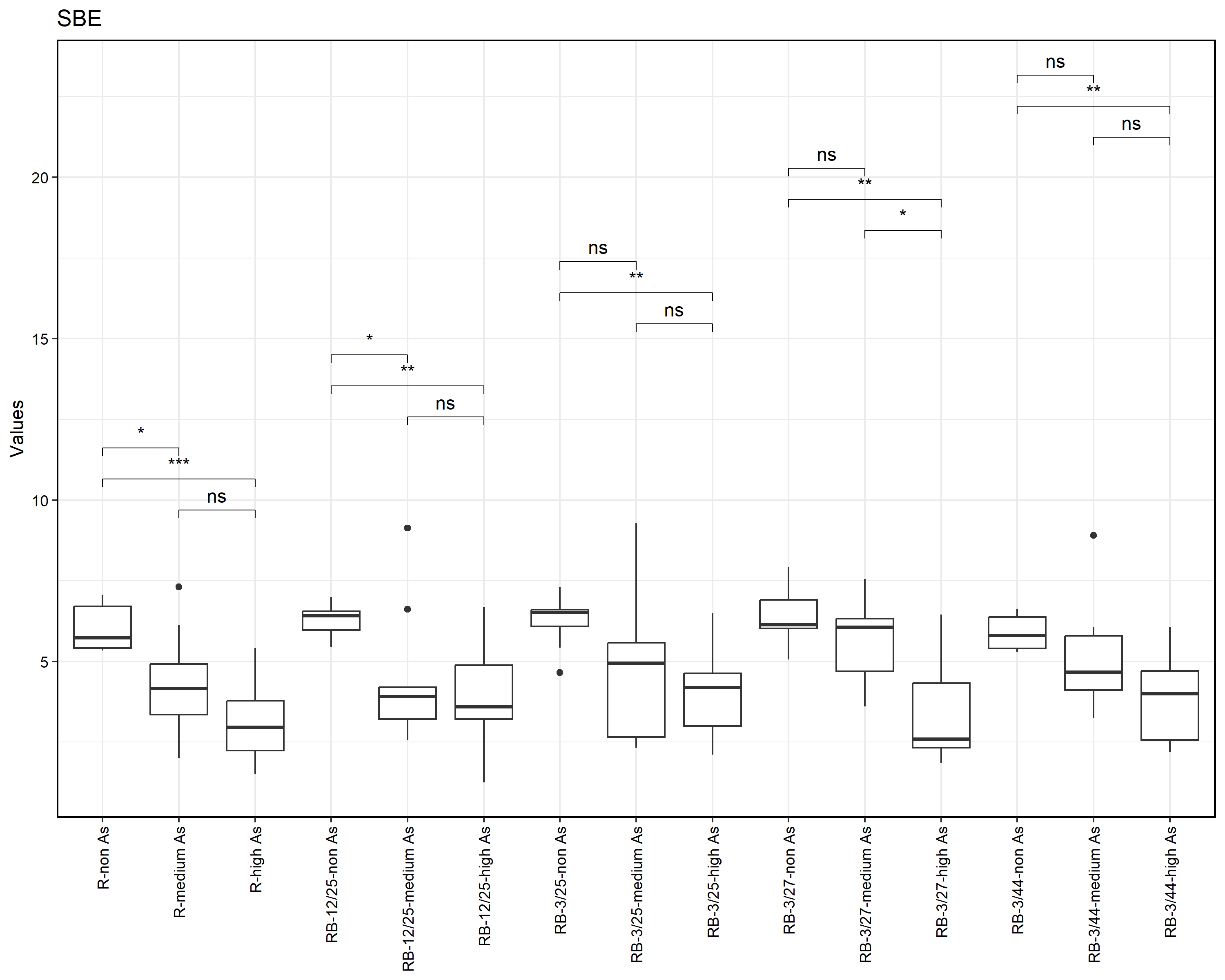 |
|  |
| 1. SDBE activity |
| 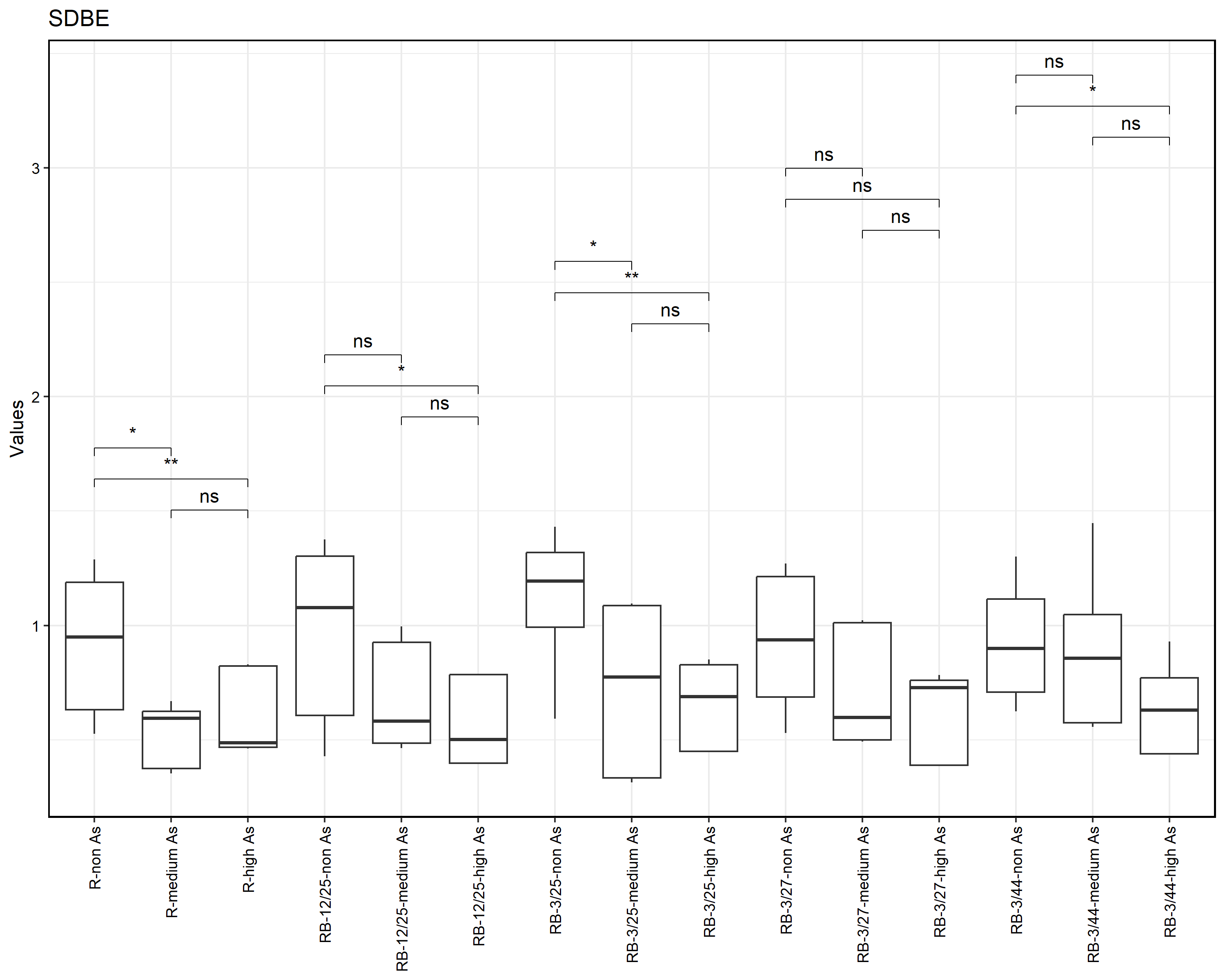 |
|  |
| 1. Starch content |
| 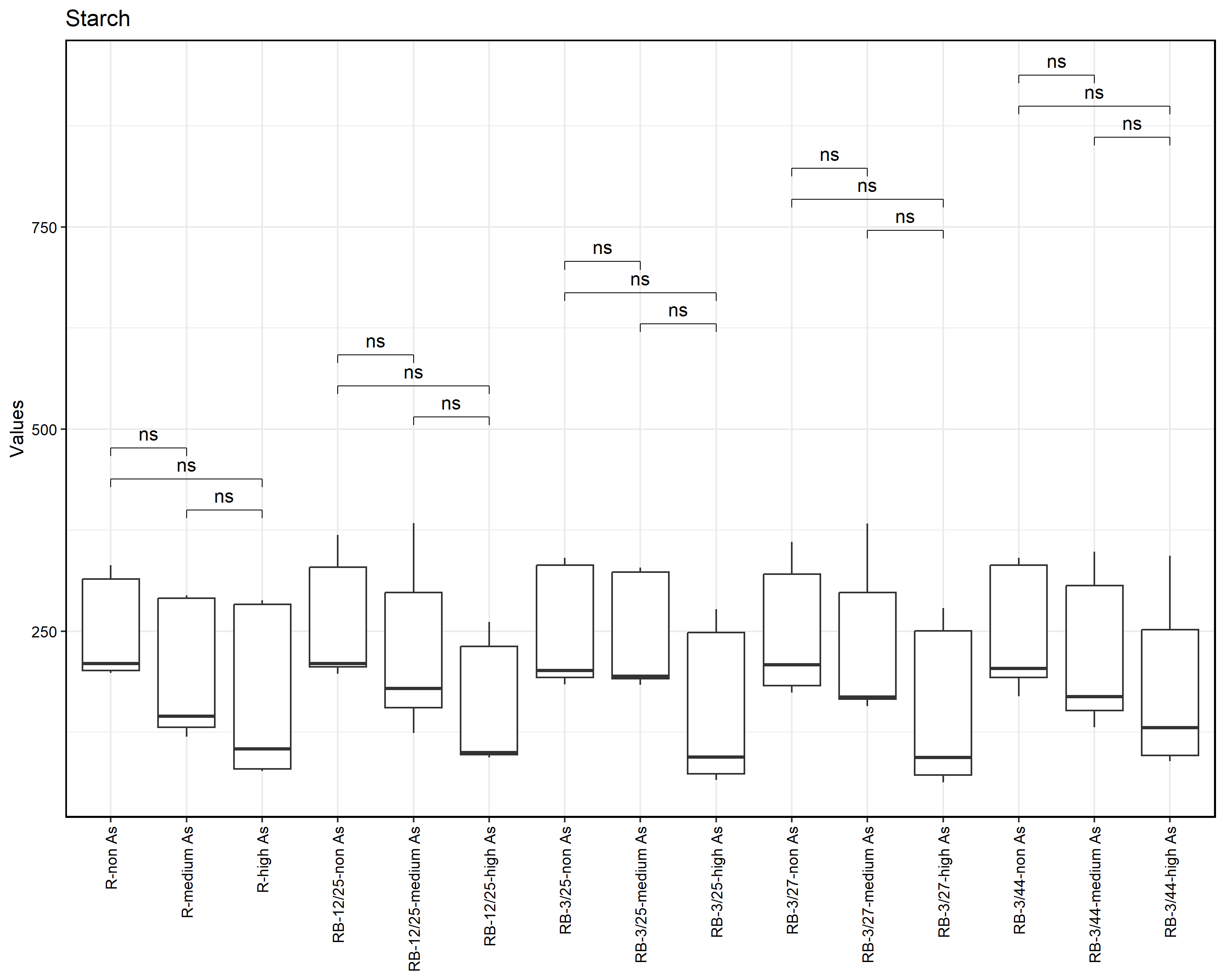 |
|  |
| 1. Amylose content |
| 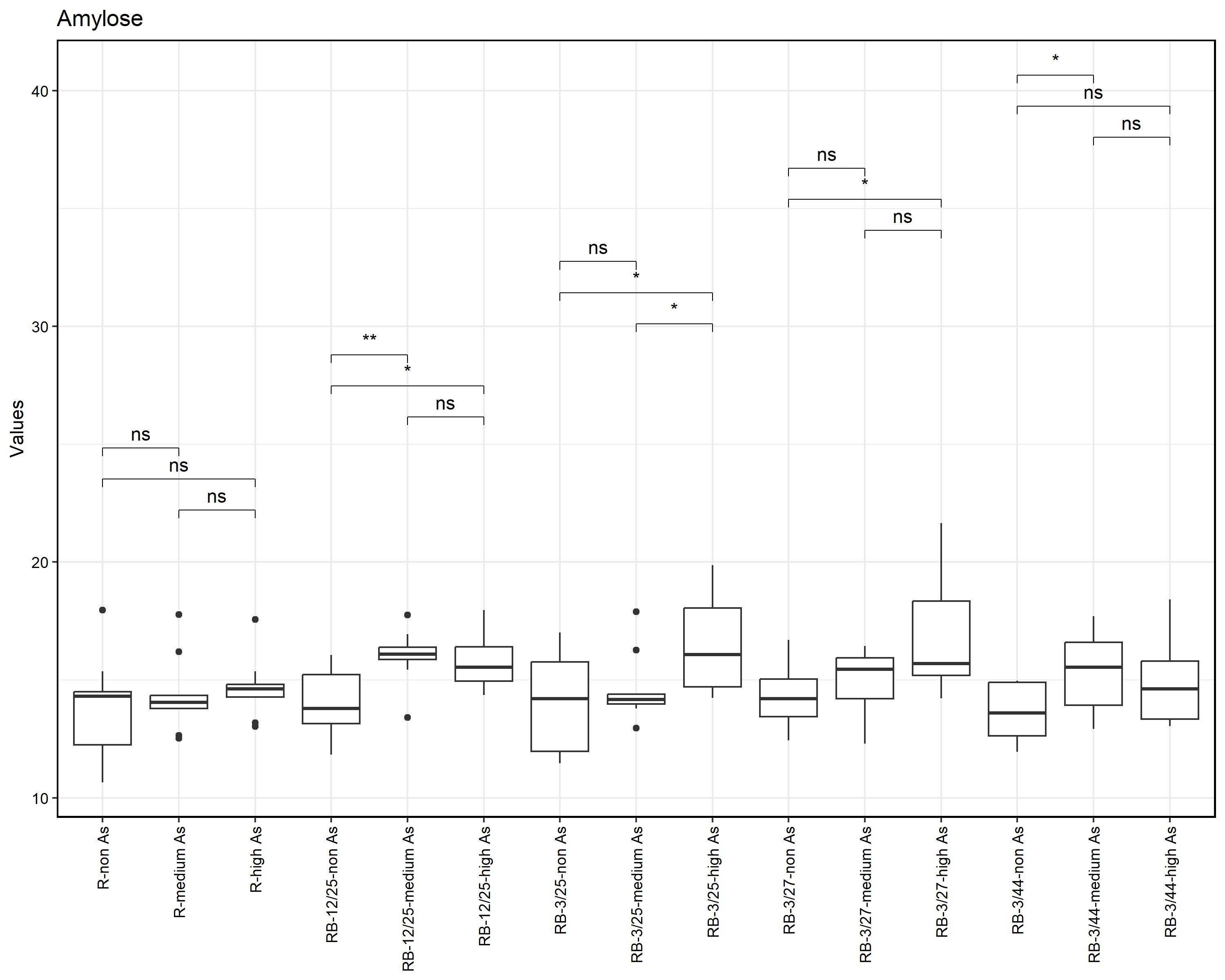 |

**Figure S1** Schematic of pairwise comparison of starch synthesis-related enzyme activities, including (a) AGPase, (b) GBSS, (c) SSS, (d) SBE, and (e) SDBE, (f) starch content, and (g) amylose content using Wilcoxon signed rank test of 15 sub-groups based on bacterial combination treatment and the concentration of arsenic (As) (5 combination treatments x 3 As levels in soils). Statistical significant symbols of p-values:  **** <0.0001, *** <0.001, ** <0.01, * <0.05, ns >0.05

| 1. AGPase activity |
| --- |
|  |
| 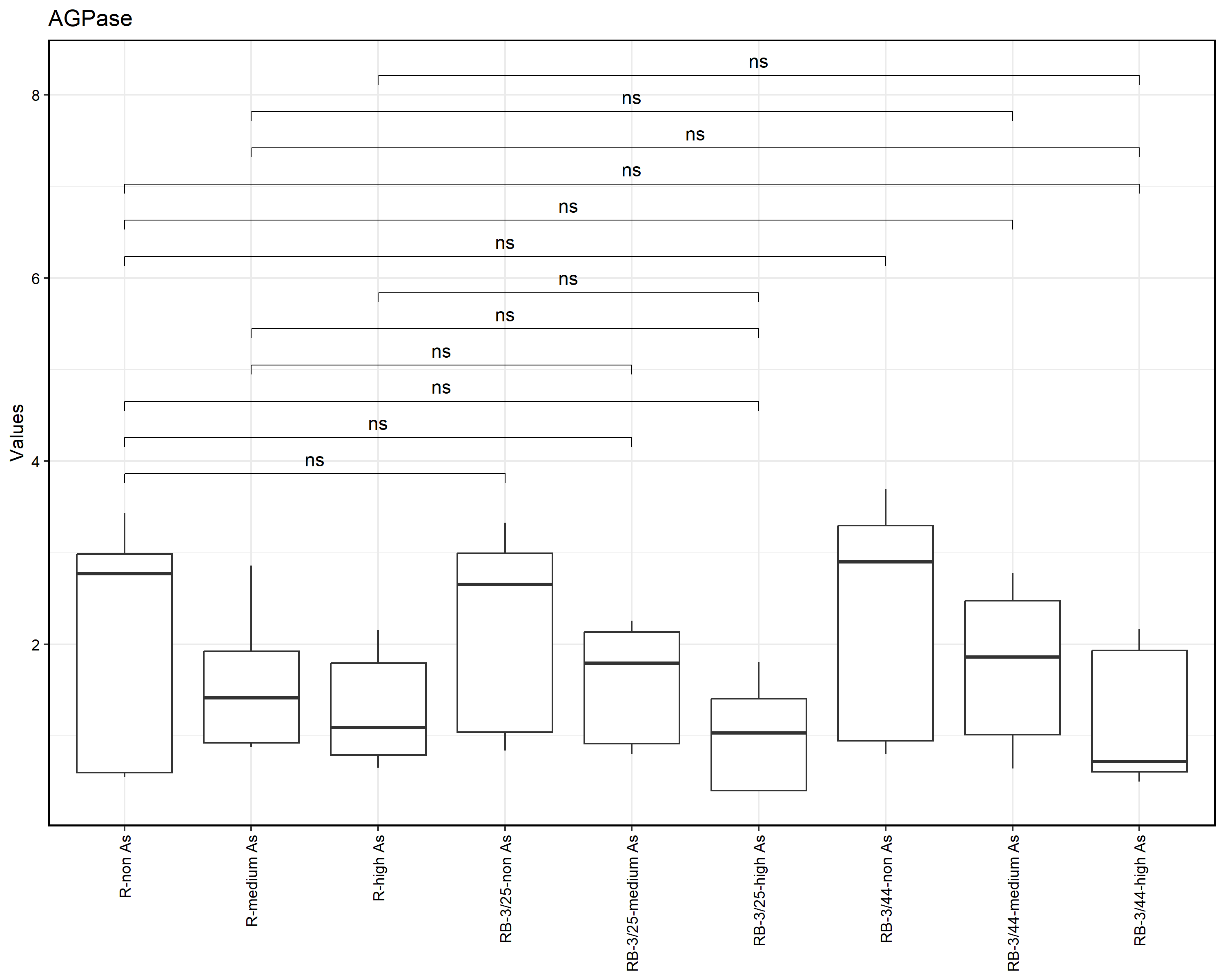 |
| (b) GBSS activity |
|  |
| 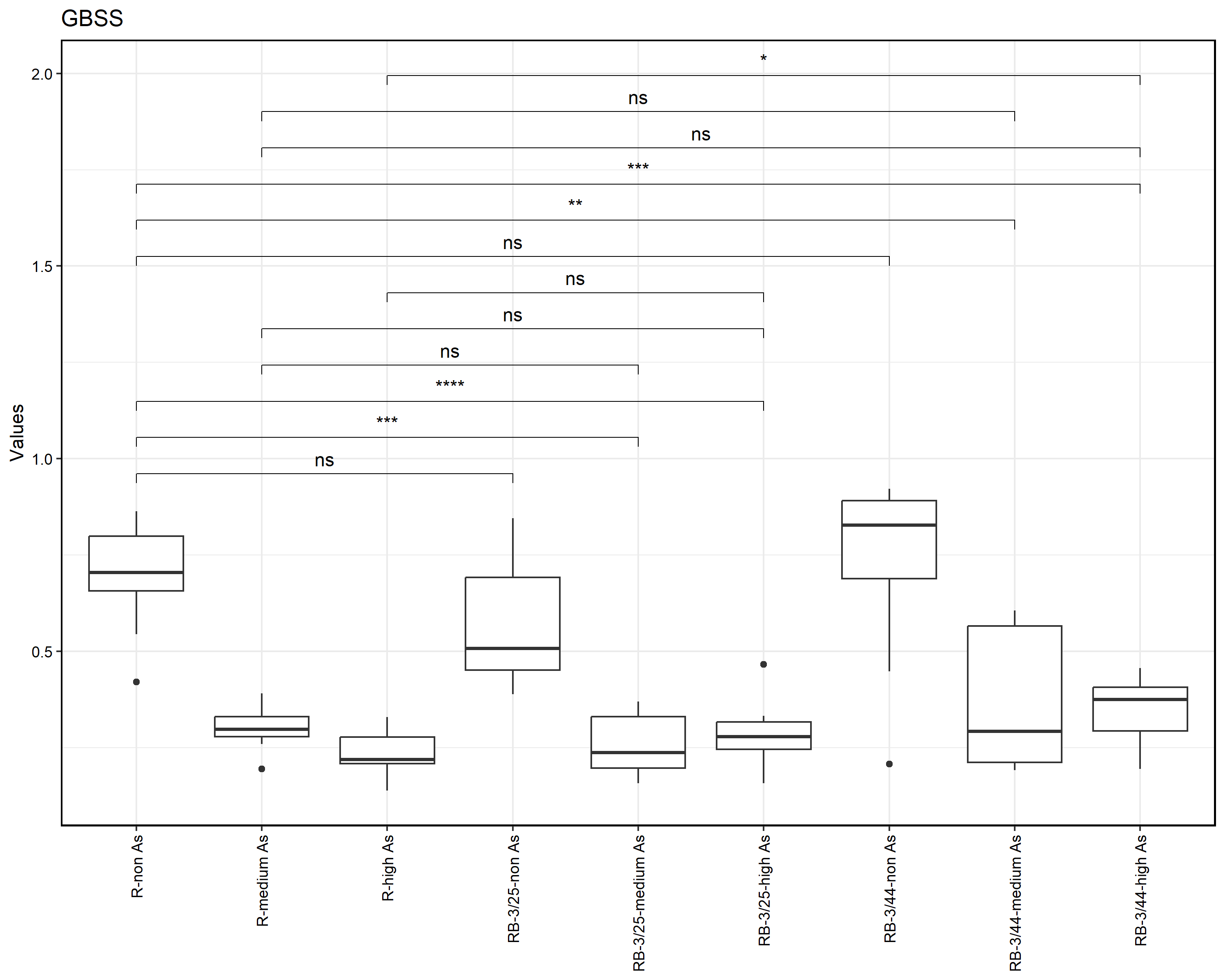 |
| (c) SSS activity |
|  |
| 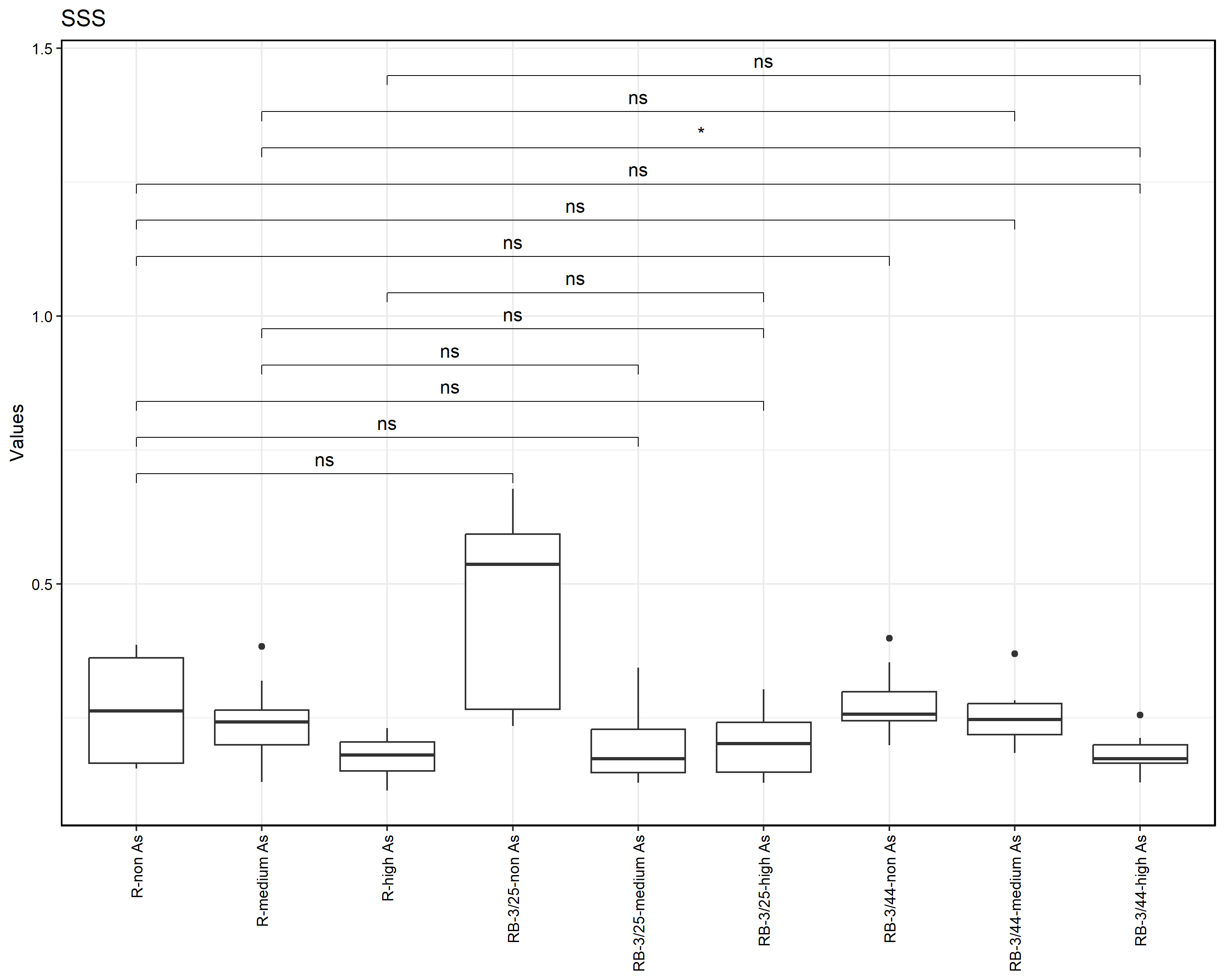 |
| (d) SBE activity |
|  |
| 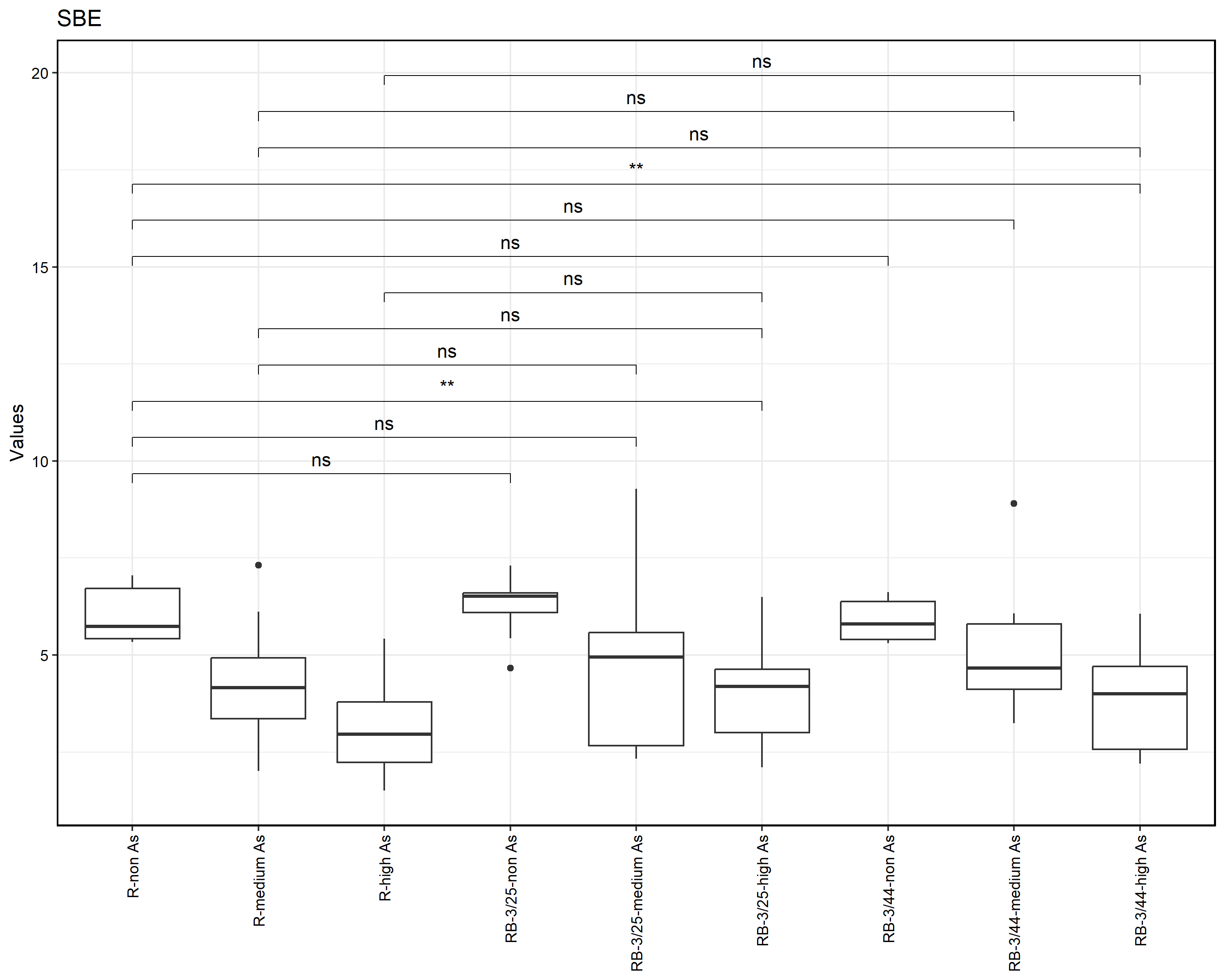 |
| (e) SDBE activity |
|  |
| 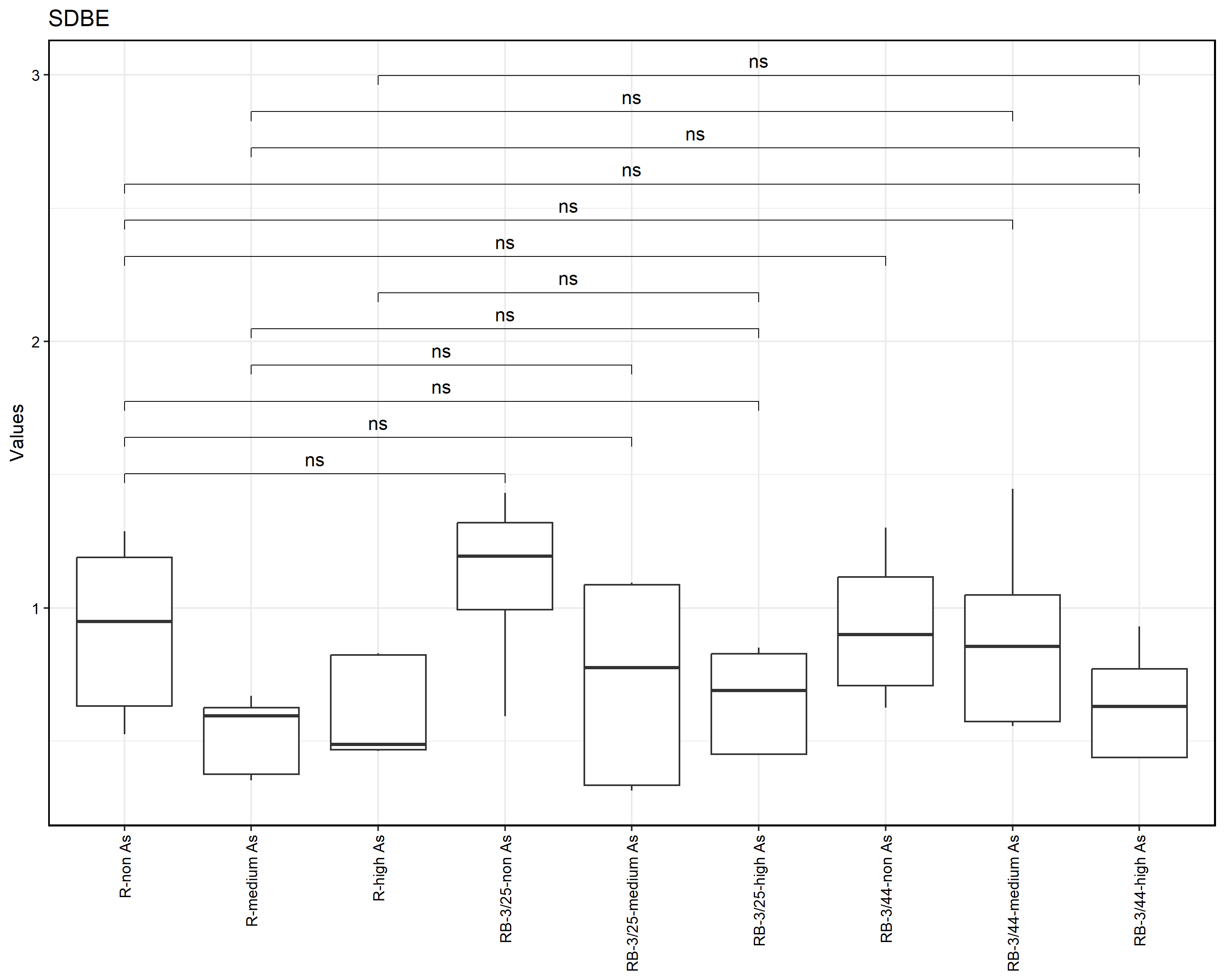 |
| (f) Starch content |
|  |
| 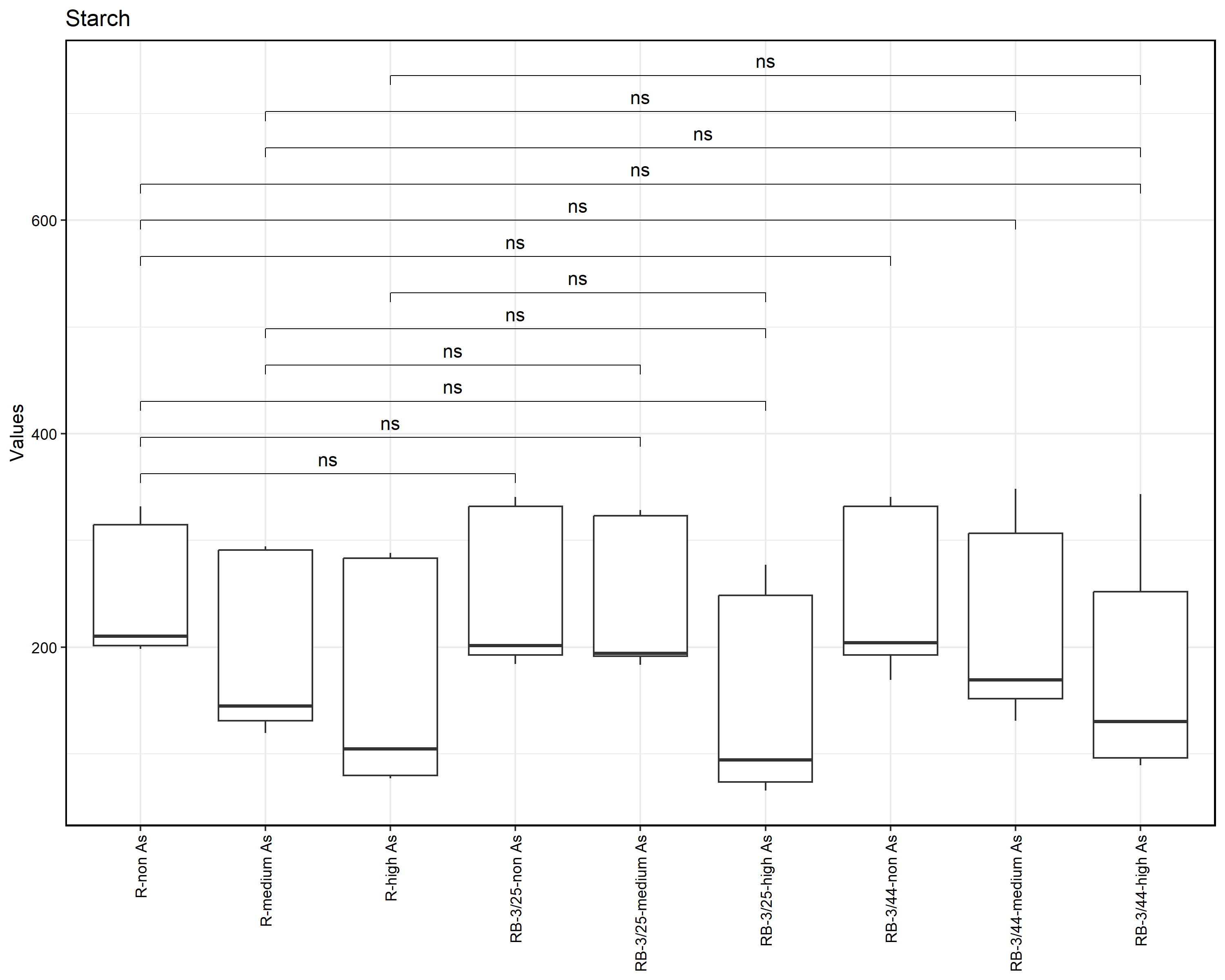 |
| (g) Amylose content |
|  |
| 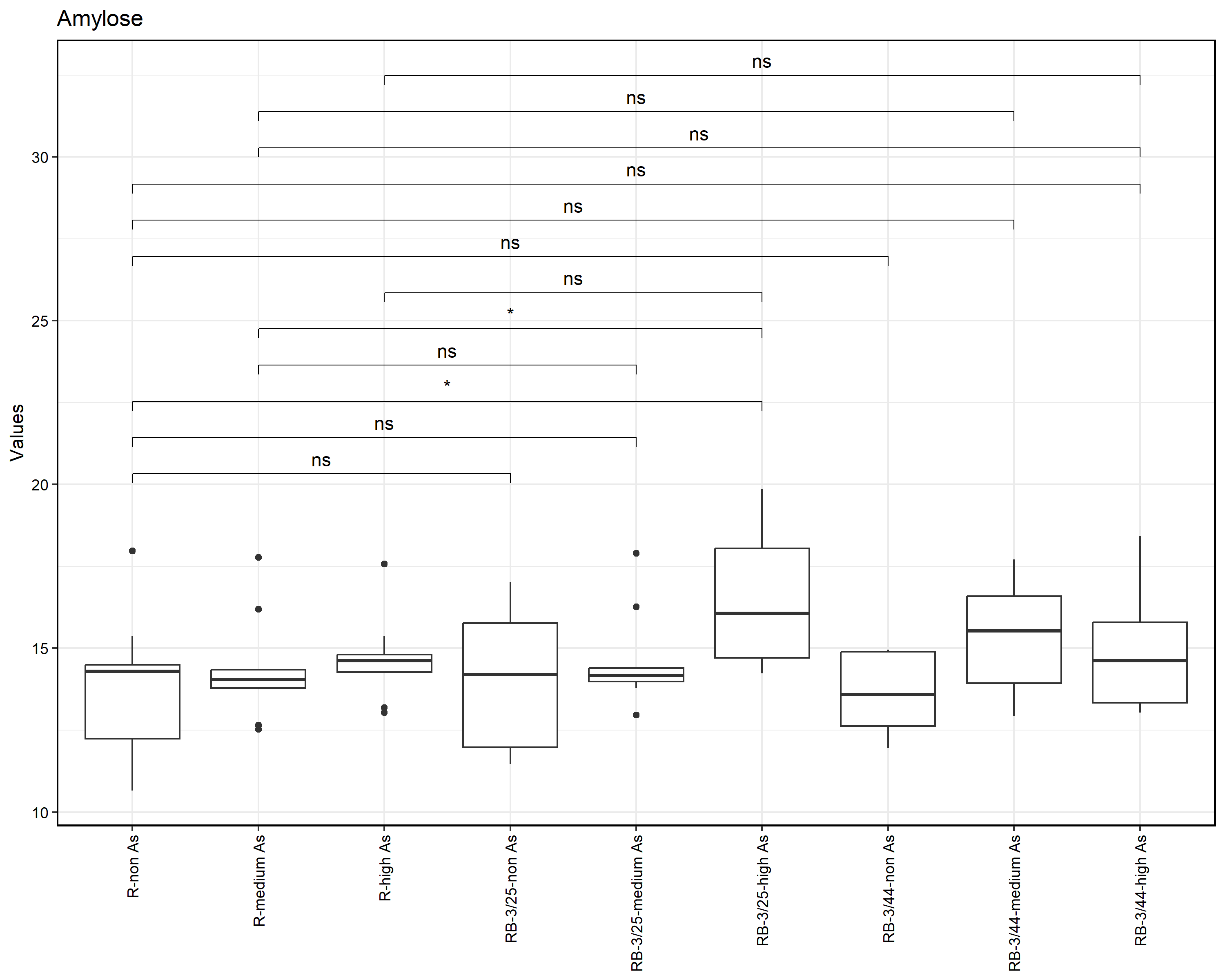 |

**Figure S2** Schematic of pairwise comparison of starch synthesis-related enzyme activities, including (a) AGPase, (b) GBSS, (c) SSS, (d) SBE, and (e) SDBE, (f) starch content, and (g) amylose content using Wilcoxon signed rank test of 15 sub-groups based on bacterial combination treatment and the concentration of arsenic (As) (5 combination treatments x 3 As levels in soils). Statistical significant symbols of p-values:  **** <0.0001, *** <0.001, ** <0.01, * <0.05, ns >0.05

**Table S1** Chemical and physical properties of the experimental soils

| **Chemical and physical properties** | **Non-As contaminated soil** | **Medium-As contaminated soil** | **High-As contaminated soil** |
| --- | --- | --- | --- |
| **Physical properties** |  |  |  |
| Sand (%) | 56.00 | 55.93 | 49.93 |
| Silt (%) | 42.00 | 43.07 | 23.93 |
| Clay (%) | 2.00 | 1.00 | 26.64 |
| Texture class | Sandy loam | Sandy loam | Sandy clay loam |
| **Chemical properties (USDA system)** |  |  |  |
| pH (1:1 H_2_O) | 7.01 | 6.40 | 5.01 |
| Organic matter (%) | 3.11 | 2.43 | 3.37 |
| Total nitrogen (mg/kg) | 1,040.00 | 969.00 | 1,580.96 |
| Total phosphorus (mg/kg) | 269.75 | 387.40 | 256.38 |
| Total potassium (mg/kg) | 3,974.00 | 5,168.43 | 5,505.42 |
| Available phosphorus (mg/kg) | 45.13 | 12.40 | 4.71 |
| Exchangeable potassium (mg/kg) | 256.66 | 109.47 | 101.42 |
| Electrical conductivity (EC: dS/m) | 0.215 | 0.162 | 0.276 |
| Cation exchange capacity (CEC) (c mol/kg) | 22.09 | 19.79 | 10.56 |
| Exchangeable calcium (mg/kg) | 4,277.00 | 2,982.00 | 1,240.00 |
| Exchangeable sodium (mg/kg) | 160.80 | 144.12 | 61.63 |
| Exchangeable magnesium (mg/kg) | 382.84 | 248.43 | 181.92 |
| Exchangeable zinc (mg/kg) | 2.47 | 1.54 | 4.24 |
| Exchangeable SO_4_^2-^ (mg/kg) | 20.50 | 82.26 | 275.25 |
| **Arsenic contents** |  |  |  |
| Total arsenic content (mg/kg) | ND | 29.13 | 45.11 |
| Available arsenic content (mg/kg) | ND | 9.13 | 21.34 |
